# Integrative genomic and transcriptomic profiles from a prospective cervical cancer study

**DOI:** 10.1038/s41698-026-01616-6

**Published:** 2026-07-22

**Authors:** Solenn Barraud, Lolita Lecompte, Rebecca Loison, Stacy Cyrille, Emmanuelle Jeannot, Elodie Girard, Sylvain Baulande, Patricia Legoix, Abderaouf Hamza, Fabrice Lecuru, Léa Pauly, Constance Lamy, Célia Dupain, Olfa Chouchane-Mlik, Alexandra Oniga, Michel Mittelbronn, Christophe Le Tourneau, Aurélien Latouche, Nicolas Servant, Ivan Bièche, Suzy M. Scholl, Maud Kamal

**Affiliations:** 1 Department of Drug Development and Innovation (D3i), Institut Curie, PSL Research University, Paris, France; 2 INSERM U1331 Computational oncology, PSL Research University, Mines Paris Tech, Paris, France; 3 Statistical Methods for Precision Medicine (STAMPM), INSERM, U1331, Computational oncology, PSL Research University, Saint-Cloud, France; 4 Department of Genetics, Institut Curie, PSL Research University, Paris, France; 5 Department of Pathology, Institut Curie, PSL Research University, Paris, France; 6 Institut Curie, Genomics of Excellence (ICGex) Platform, PSL Research University, Paris, France; 7 Department of Breast, Gynecological and Reconstructive Surgery, Institut Curie, Université Paris Cité, Paris, France; 8 National Center of Pathology (NCP), Laboratoire National de Santé (LNS), Dudelange, Luxembourg; 9 Luxembourg Center of Neuropathology (LCNP), Luxembourg, Luxembourg; 10 Department of Cancer Research (DoCR); Luxembourg Institute of Health (LIH), Luxembourg, Luxembourg; 11 Department of Life Sciences and Medicine (DLSM); University of Luxembourg, Esch sur Alzette, Luxembourg; 12 Luxembourg Centre for Systems Biomedicine (LCSB), University of Luxembourg, Esch-sur-Alzette, Luxembourg; 13 Faculty of Science, Technology and Medicine (FSTM), University of Luxembourg, Esch-sur-Alzette, Luxembourg; 14 Conservatoire National des Arts et Métiers, Paris, France; 15 INSERM U1016, Paris Cité University, Faculty of Pharmaceutical and Biological Sciences, Paris, France

**Keywords:** Cervical cancer, Cancer genetics, Tumour biomarkers

## Abstract

Cervical cancer (CC) is a global public health concern, with frequent relapses and limited therapeutic options for metastatic disease. The RAIDs project, launched by the Curie Institute, aims to improve molecular characterization and risk stratification in CC. High-quality primary frozen tumor samples from 291 (WES), 302 (sWGS), and 270 (RNA-seq) patients enrolled in the Bio-RAIDs European study (NCT02428842) were analyzed alongside clinical data. Frequent alterations included *PIK3CA* (30%), *KMT2D* (11%), and *KMT2C* (11%). Only *TERT* mutations (13%) were significantly associated with poor progression-free survival (PFS). Targetable mutations in *PIK3CA* (30%), *KRAS* (4%), *ERBB2* (4%), and *FGFR3* fusions (5%), were found in 57% of patients. Transcriptomic analysis identified *TERT* and the Senescence pathway as significantly linked to PFS and to necrosis. Gene expression deconvolution showed no significant variation across immune populations. Integrative likelihood-based boosting analysis of 234 patients identified *GNAQ* and *THUMPD1* as protective factors for PFS, while necrosis, age >50 years, advanced FIGO stage (III/IV), and HPV-negative status were associated with significantly poorer outcomes. These integrative multi-omics analyses revealed novel prognostic biomarkers in CC and identified over 50 clinically actionable mutations. Transcriptomic profiles were associated with both genetic alterations and histopathological features, suggesting new paths for precision medicine in CC.

## Introduction

Although advancements have been made in the accurate staging and management of cervical cancer (CC), the risk of recurrence remains considerable, particularly in patients with locally advanced disease at the time of diagnosis, which constitutes most patients referred to clinical and radiation oncologists. A deeper understanding of the biology of each tumor is crucial for stratifying the risk of recurrence following standard therapy and for directing the management of patients. Despite the availability of effective prophylactic vaccines against the human papillomavirus (HPV), CC remains the fourth most prevalent cancer globally^1^.

Management of cervical cancer is categorized into surgical approaches for early-stage disease and chemoradiation involving a platinum-based regimen followed by brachytherapy for locally advanced or advanced cases; however, numerous variations are noted, as previously published, due to lymph nodes identified in surgical resection specimens or variations in treatment tolerance. Despite optimal management, disseminated or recurrent disease cannot currently be treated with curative intent, with a reported risk of relapse nearing 50% in cases of locally advanced disease^2–4^. Immunotherapy involving immune checkpoint inhibitors (ICIs) has recently emerged as a therapeutic option for metastatic disease, either as a standalone treatment or in combination with systemic chemotherapy. The prescription of ICIs is contingent upon a CPS score (a combined positive score based on PDL-1 labelling via immunohistochemistry (IHC)), which must be equal to or greater than 1 to qualify for health insurance reimbursement. Notably, large, randomized phase III trials have thus far demonstrated very limited efficacy of ICIs, resulting in a median progression-free survival of approximately 3 months with monotherapy^5^. Various other therapies, including vascular supply inhibitors such as the VEGF inhibitor bevacizumab^6^, have shown minimal effects, and many are currently undergoing clinical trials. Therapeutic vaccination strategies utilizing viral vectors for HPV-induced cancers are presently under evaluation in phase I/II studies.

Bio-RAIDs (NCT02428842) was a prospectively collected CC dataset (*n* = 419), which formed part of the EU-funded project “RAIDs” (Rational Molecular Assessments and Innovative Drug Selection). The objective of this project was to develop innovative tools, models, and new standards for CC treatment. The consortium previously reported an analysis on half of the RAIDs population. The most prevalent oncogenic alterations were *PIK3CA* (42%), which was consistent with previous literature^7^. Furthermore, a loss of function in epigenetic regulators in conjunction with driver alterations in the *PIK3CA* pathway was found to be correlated with poor PFS^8^. Subsequently, a multi-omics approach was undertaken utilizing 89 patients for whom analysis of WES, a DNA panel of 608 genes, CNV, as well as RPPA were available. The post-translational modification of β-catenin at Serine 552 (β-Cat-Ser552) was found to be significantly associated with PFS^9^. Finally, the consortium demonstrated that the presence of necrosis was a negative prognostic factor significantly associated with lower progression-free survival^10^. Here, we sought to report extensive molecular analyses of CC patients included Bio-RAIDs.

## Results

### Genomic landscape of early-stage/locally advanced CC

For all patients with PFS data and for whom quality-controlled primary frozen tumor samples were available, WES, sWGS and RNA sequencing were performed in 291, 302 and 270 patients, respectively, in a single-center analysis.

**Fig. 1:**
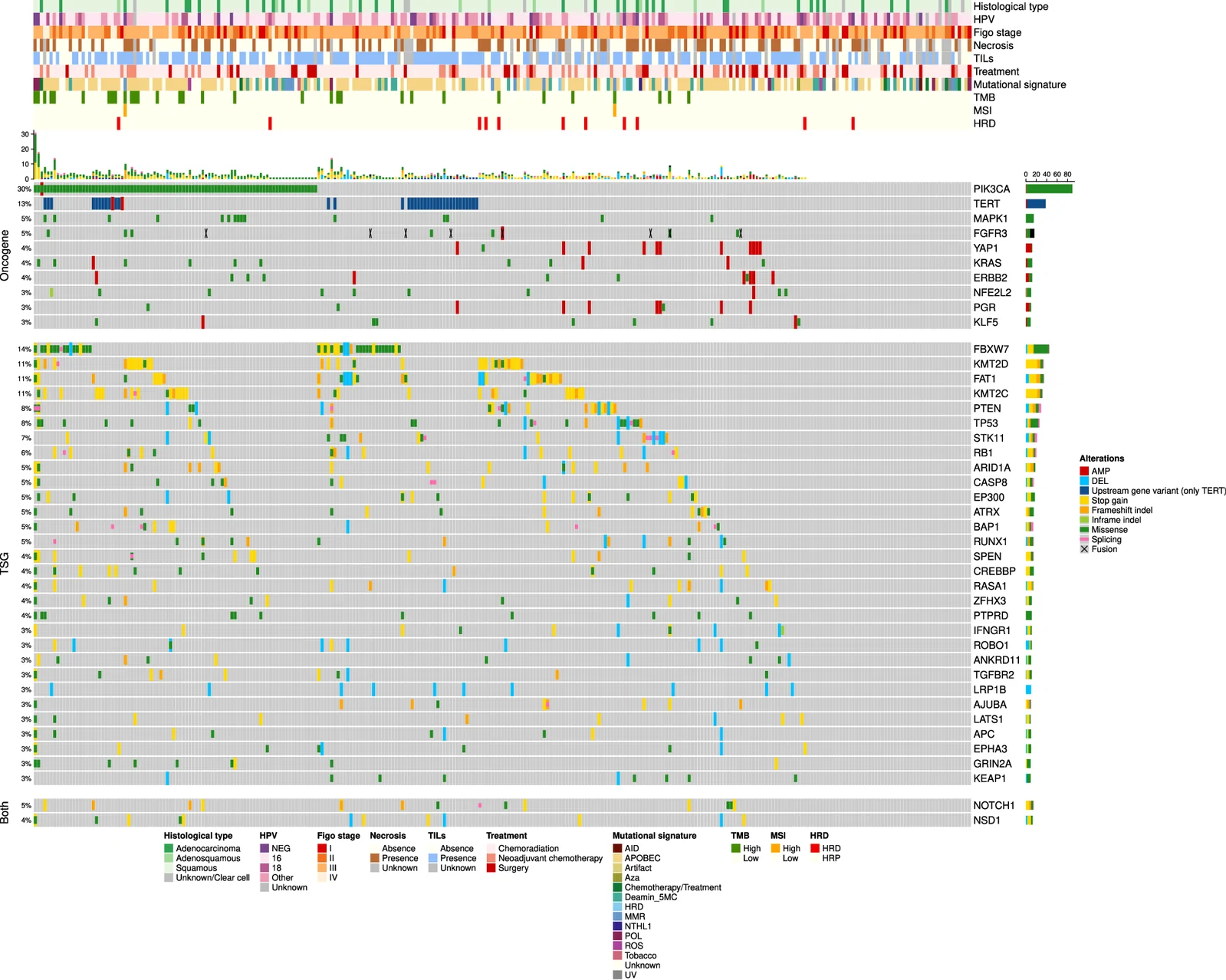
Oncoprints presenting an overview of the most frequently altered (≥3%) oncogenes and tumor suppressor genes. Neg = negative, TILSs = Tumor-Infiltrating Lymphocytes, TMB = Tumor Mutational Burden, MSI = Microsatellite Instability, HRD = Homologous Recombination Deficiency, Borderline = Borderline samples for HRD status, HRP = Homologous Recombination Proficiency, AMP = Amplifications, DEL = Deletion.

Demographic, clinical, and treatment data are shown in Table 1. Genomic alterations of locally advanced CC are represented for each patient in Fig. 1 and include somatic single nucleotide variants (SNVs), short indels, copy number alterations (CNVs), fusion genes, along with genomic signatures such as the tumor mutational burden (TMB), microsatellite instabilities (MSI), mutational signatures, homologous recombination deficiencies (HRD), together with clinical data. The most frequently altered oncogenes were *PIK3CA* (30%), *TERT* (13%), *MAPK1* (5%), *FGFR3* (5%), *YAP1* (4%), *KRAS* (4%), *ERBB2* (4%), *NFE2L2* (3%), *PGR* (3%), and *KLF5* (3%). A box plot analysis in Supplementary Fig. 4 demonstrates differential expression levels of *TERT* in CC, according to the presence (mutated) or absence (wild type) of hotspot promoter mutations, with a significant increase in *TERT* expression in samples harboring upstream mutations (*p*-value = 1.2 × 10^−^^7^, Wilcoxon-Mann-Whitney test). The most frequently altered tumor-suppressor genes were *FBXW7* (14%), *KMT2D* (11%), *FAT1* (11%), *KMT2C* (11%), *PTEN* (8%), *TP53* (8%), *STK11* (7%), *RB1* (6%), *ARID1A* (6%), *CASP8* (5%), *EP300* (5%), *ATRX* (5%), *BAP1* (5%) and *RUNX1* (5%) (Supplementary Data 1). Most frequently reported gene fusions were *FGFR3* (*n* = 8, 5%) and *FGFR2* (*n* = 1, 0.3%) (Supplementary Data 2). Thirty-two tumors (11%) revealed a TMB-high (>10) score; two of which were also MSI-high (Supplementary Data 3).

**Table. 1:** Clinical and biological characteristics of BioRAID’s patients with Cervical Cancer (CC)

	Clinical dataset	WES dataset	sWGS dataset	RNA-Seq dataset	Complete dataset
	*N* = 377	*N* = 291	*N* = 302	*N* = 270	*N* = 266
Age in years					
≤50	184 (49%)	140 (48%)	147 (49%)	129 (48%)	127 (48%)
>50	193 (51%)	151 (52%)	155 (51%)	141 (52%)	139 (52%)
2018 FIGO Staging Classification for Cervical Cancer					
FIGO stage I/II	137 (36%)	95 (33%)	98 (32%)	85 (31%)	84 (32%)
FIGO stage III/IV	240 (64%)	196 (67%)	204 (68%)	185 (69%)	182 (68%)
Histology					
Squamous	309 (82%)	237 (81%)	246 (81%)	221 (82%)	218 (82%)
Adenocarcinoma	43 (11%)	35 (12%)	37 (12%)	34 (13%)	33 (12%)
Adenosquamous	15 (4%)	10 (3%)	10 (3.3%)	8 (3%)	8 (3.0%)
Mixed/undifferentiated/clear cell/other	10 (3%)	9 (3%)	9 (3.0%)	7 (3%)	7 (2.6%)
HPV negative					
No	309 (93%)	263 (93%)	274 (94%)	247 (94%)	243 (93%)
Yes	23 (7%)	19 (7%)	19 (6.5%)	17 (6%)	17 (7%)
Unknown	45	9	9	6	6
Necrosis					
No	192 (65%)	162 (64%)	169 (65%)	155 (65%)	153 (65%)
Yes	105 (35%)	92 (36%)	93 (35%)	85 (35%)	83 (35%)
Unknown	80	37	40	30	30
Initial Treatment					
Chemoradiation	263 (70%)	203 (70%)	211 (70%)	190 (70%)	187 (70%)
Neoadjuvant chemotherapy	58 (15%)	48 (16%)	49 (16%)	44 (16%)	43 (16%)
Surgery	56 (15%)	40 (14%)	42 (14%)	36 (13%)	36 (14%)
PFS Event	120 (32%)	99 (34%)	100 (33%)	93 (34%)	91 (34%)
TILs					
Absence/No (<= 10)	104 (28%)	87 (30%)	88 (29%)	79 (29%)	78 (29%)
Presence/Yes (> 10)	192 (51%)	166 (57%)	173 (57%)	160 (59%)	157 (59%)
Unknown	81	38	41	31	31

*NACT* Neoadjuvant chemotherapy, *NA* not available.

Supplementary Data 4 shows the most contributing mutational signature for each tumor. Eleven (11/302) (4%) had a homologous repair deficiency (HRD) status, while most (96%) were classified as homologous repair proficient (HRP) (Supplementary Data 5). Three (3/11) tumors had mutations in one of the homologous recombination genes: one *BRCA1* mutation, one *BRCA2* mutation, and one *BARD1* mutation (Supplementary Data 6). We also investigated germline mutations in homologous recombination (HR) genes. We identified 15 germline pathogenic or likely pathogenic variants in 14 patients **(**Supplementary Data 7**)**.

When assessing DNA alterations by biological functions and cellular pathways (Supplementary Fig. 5, Supplementary Data 8), recurrently altered signaling pathways and cellular processes were revealed. Pathway-based analysis of 709 altered genes according to the OncoKB precision algorithm showed these to be preferentially in the PI3K (49%), Epigenetics (43%), RTK/RAS (39%), Transcription-factor regulator (36%), DNA-repair (26%), Development and Notch (23%), Hippo (22%), Regulation of Gene Expression (21%), Cell Cycle (16%), Immunity (15%), Apoptosis (14%), Senescence (14%), Wnt (12%) and MAPK (12%) signalling pathways. Stratifying frequently altered genes according to the OncoKBclassification^11^, on clinical actionability (Supplementary Fig. 6), the most targetable genes were *PIK3CA* (30%), *PTEN* (8%), *FGFR3* (5%), *BRCA2* (4%), *KRAS* (4%), and *ERBB2* (4%).

### Transcriptomic landscape of both early stage or locally advanced CC

**Fig. 2:**
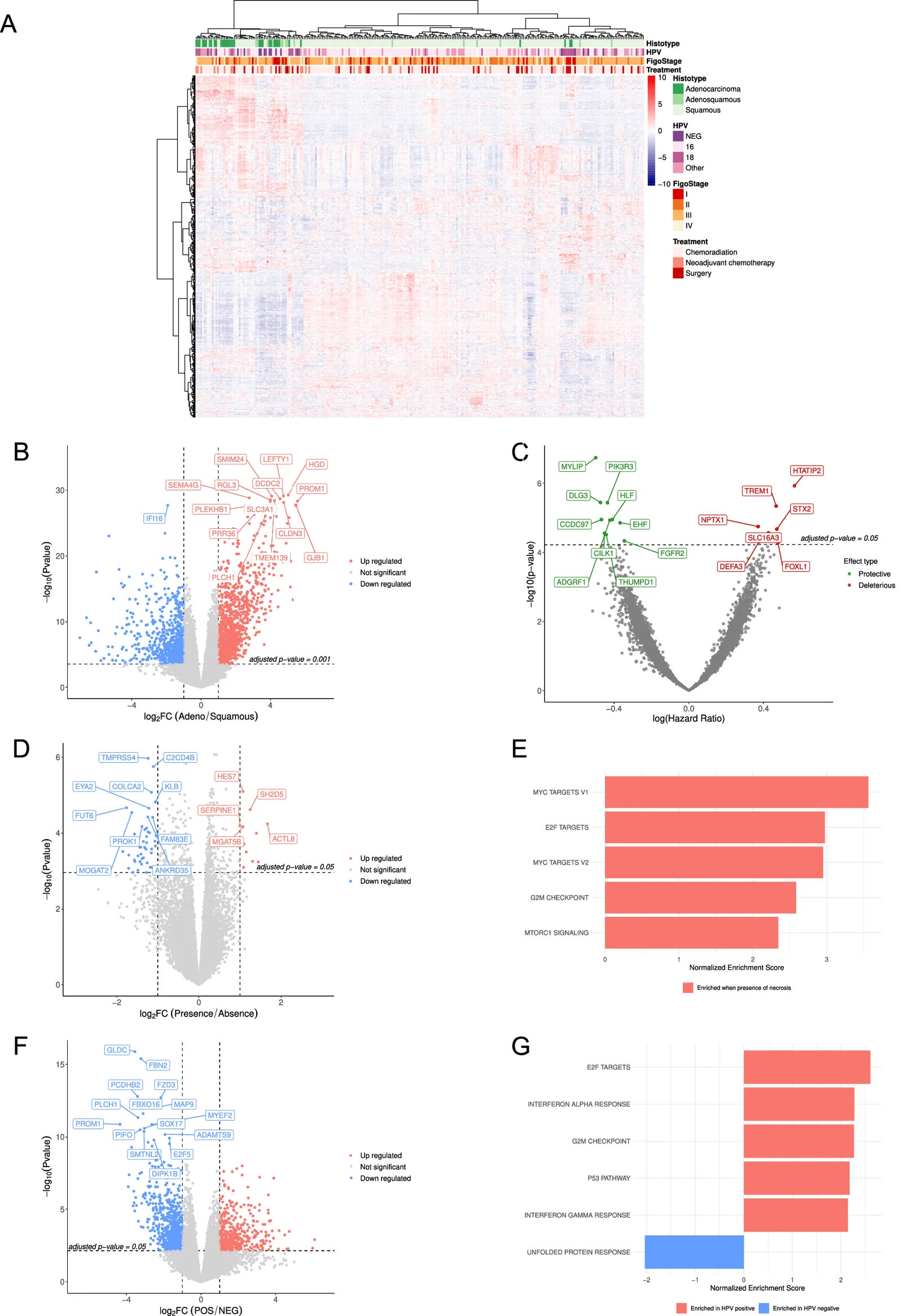
Transcriptomic analyses according to clinicopathological features and patient outcome. **A** Hierarchical clustering based on the 1000 most varying genes. **B** Volcano plot representing differentially expressed genes according to the histological subtype: Adenocarcinoma and adenosquamous tumors versus Squamous-cell carcinomas (adjusted *p*-value = 0.001). **C** Volcano plot representing differentially expressed genes associated with the Progression Free Survival using a Cox proportional hazard model, x-axis represents log(Hazard ratio) and y-axis represents –log10(Log-rank *p*-value) (adjusted *p*-value = 0.05). **D** Volcano plot representing differentially expressed genes according to the presence of necrosis (adjusted *p*-value = 0.05) **E** Significantly enriched Hallmark gene sets from Gene Set Enrichment Analysis according to the presence or absence of necrosis using MSigDB database (adjusted *p*-value < 0.001, |NES | >2) **F** Volcano plot representing differentially expressed genes according to HPV status (positive versus negative) (adjusted *p*-value = 0.05) **G** Significantly enriched Hallmark gene sets from Gene Set Enrichment Analysis according to HPV status (positive versus negative) using MSigDB database (adjusted *p*-value < 0.001, |NES | >2).

RNA-sequencing data of tumor samples from 270 patients first underwent unsupervised hierarchical clustering based on the 1000 most highly variable genes (Fig. 2A). In order to verify that our population was truly representative of CC, we initially conducted a supervised clustering based on the histological type. As expected, we highlighted differences in expression between squamous cell carcinomas on one hand and adenocarcinomas and adenosquamous carcinomas on the other (Fig. 2B**)**. A Cox proportional hazards regression model was used to identify genes with expression levels associated with progression-free survival (PFS). Several genes showed significant survival protective effects (log(HR) < 0), these included gene expression variability in the following: *MYLIP*, *PIK3R3*, *DLG3*, *CCDC97*, *HLF*, *EHF*, *CILK1*, *ADGRF1*, *THUMPD1*, and *FGFR2*, highlighted in green (Supplementary Fig. 7). Conversely, genes exhibiting a significant adverse impact on survival (log(HR) > 0), shown in red, included *HTATIP2, TREM1, STX2, NPTX1, SLC16A3, FOXL1*, and *DEFA3*. (Fig. 2C). Gene expression according to the presence or absence of necrosis in the tissue biopsy at baseline is documented in Fig. 2D^10^. *HES7* and *GAL7* were significantly enriched in presence of necrosis (adjusted *p*-value < 0.05) while *TMPRSS4*, *C2CD4B*, *EYA2*, *COLCA2*, *FUT6*, *MOGAT2*, *FAM83E* and *KLB* were significantly down-regulated. Using the MSigDB Hallmark gene set collection, significantly enriched pathways related to the presence of necrosis were the following: MYC Targets, E2F targets, G2M checkpoint and mTORC1 signaling (adjusted *p*-value < 0.001) (Fig. 2E). Finally, we compared gene expression data according to the HPV status (positive versus negative) as illustrated in Fig. 2F. *GLDC, FBN2, PCDHB2, PFXO16, PLCH1, FZD3, MAP9, MYEF2, PROM1, SOX17, E2F5, ADAMTS9, PIFO, DIPK1B* and *SMTNL2* were significantly down regulated in HPV-positive tumors (adjusted *p*-value < 0.05). Using the MSigDB Hallmark gene set collection, significantly enriched pathways associated with the HPV-positive status were the following: E2F targets, Interferon Alpha Response, G2M Checkpoint, P53 pathway and Interferon Gamma Response, while Unfolded Protein Response was significantly enriched in HPV-negative tumors (adjusted *p*-value < 0.001), Fig. 2G.

### Transcriptomic-based immune landscape of locally advanced CC

In order to assess the immune landscape of CC, we applied computational deconvolution methods to sort expression data of immune cell populations from bulk transcriptome data. Using immune cell-specific signatures (CIBERSORT^12^ we were able to infer the overall immune infiltrate and relative immune cell populations in individual CC biopsies. A substantial homogeneity of the immune infiltrate-related transcripts was observed among clinical features (Supplementary Fig. 8**)**.

### Prognostic biomarkers for standard treatment in CC

**Fig. 3:**
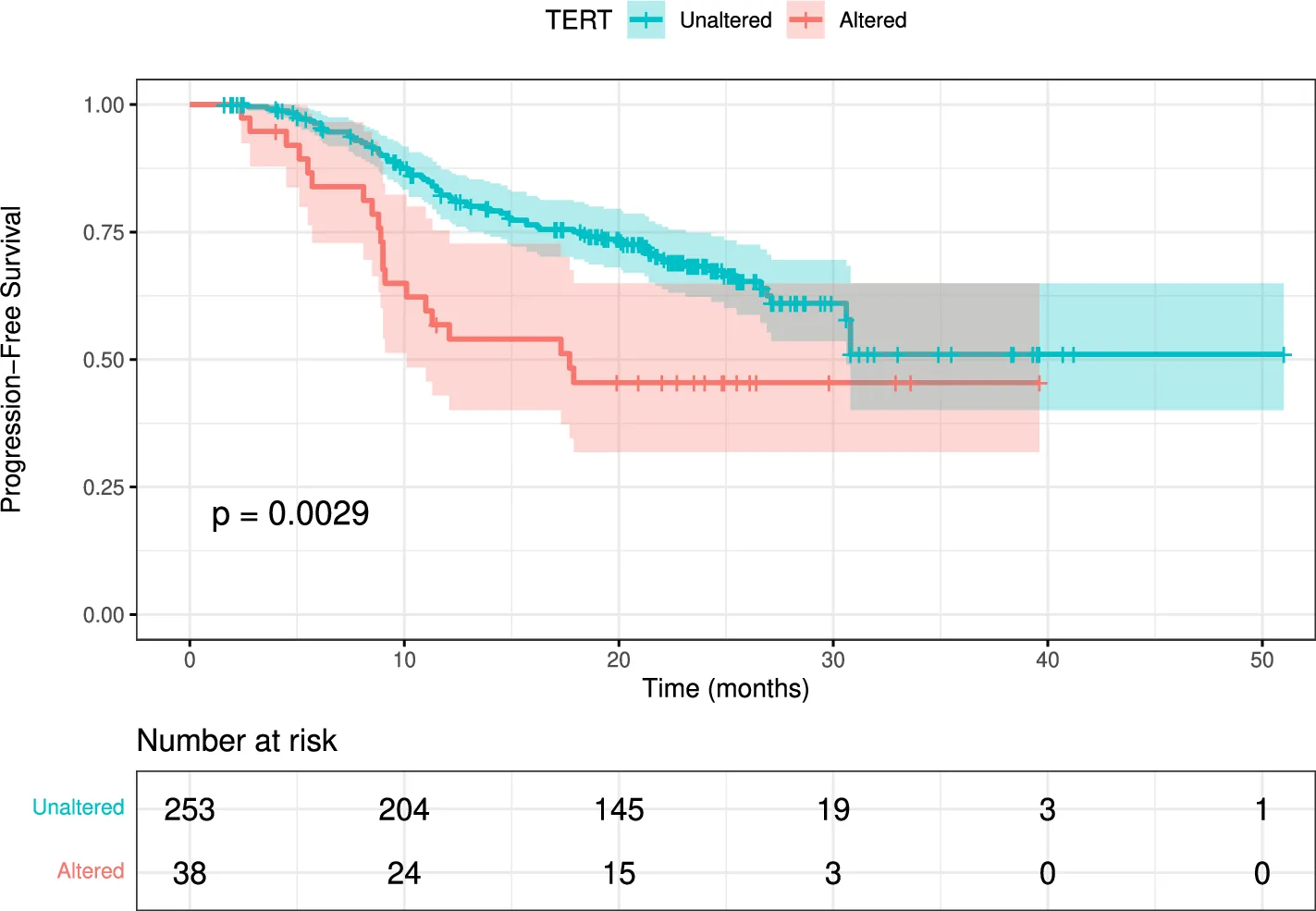
Survival curve based on Kaplan-Meier and log-rank test for *TERT* alteration.

**Fig. 4:**
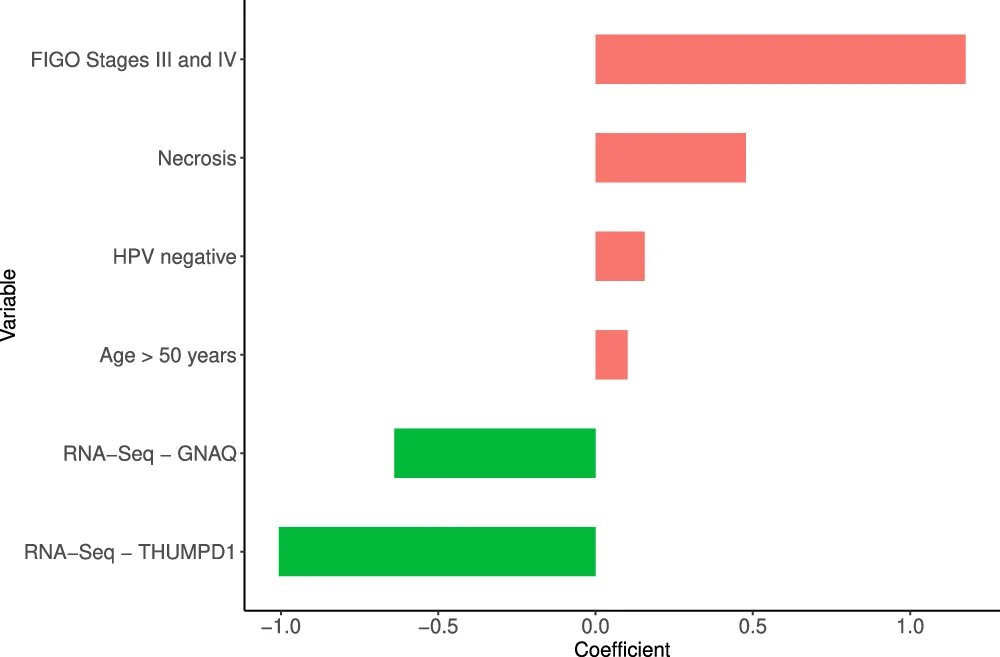
Coefficient values in Cox Boost using clinical, CNV, SNV, fusion alterations, TMB, MSI, mutational signature subgroups, HRD, and RNA-Sequencing data (*N* = 234).

We investigated the association between PFS and the identified oncogenic DNA-level alterations in conjunction with TMB or HRD status, and signaling pathway perturbations. (Supplementary Data 9 and 10). No prognostic significance was observed for TMB nor HRD while senescence pathway alterations (Supplementary Fig. 9) were significantly associated with poorer PFS (*p*-value = 0.0039, log-rank test), possibly in relation with *TERT* alterations (*p*-value = 0.0029, log-rank test) (Fig. 3). Applying a likelihood-based boosting approach on the complete cohort of 234 patients was performed, including molecular biomarkers based on DNA alterations and activated protein expression profiles together with the mandatory presence of the following clinical variables (age: ≤50 versus >50 years, 2018 FIGO stage: I/II versus III/IV, presence versus absence of necrosis and HPV status (-/+):Patients with missing assessments of either HPV or necrosis were removed from analysis. This boosting estimation included 234 patients (with all omics data available) illustrated two non-zero **negative** regression coefficients associated with *GNAQ* and *THUMPD1* (Fig. 4). High expression levels of these genes were thus associated with prolonged PFS, with *THUMPD1* showing the strongest positive impact. Necrosis had the highest **positive** coefficient, suggesting it significantly worsens PFS. Age (<50 years at diagnosis) and HPV-negative status were similarly associated with poorer outcome, with age being dominant to HPV status. In a conventional multivariable Cox regression model adjusted for these clinical covariates (necrosis in the diagnostic tumor specimen, age, FIGO stage, and HPV status), all omics features we documented remained significantly associated with PFS (Supplementary Data 11), thus highlighting the independent prognostic value of the biomarkers we identified. Our findings confirm the significant influence of both clinical features and genetic markers on the progression-free survival of CC patients.

## Discussion

Here we report on a large prospectively collected dataset from seven EU countries. Patients in 18 participating centers provided fresh-frozen, quality-controlled baseline samples of cervical cancer (CC), for which the analysis—using not only whole-exome sequencing but also shallow whole-genome and RNA sequencing—is now complete.

The Bio-RAIDs consortium previously reported molecular analysis on half (48%) of the RAIDs population. The most prevalent oncogenic alterations detected earlier in a **DNA panel of 608 genes** analyzed in a different centre and based on different technical procedures, had been *PIK3CA* (42%)^8^, higher than using WES in the present analysis but consistent with a report from the Cancer Genome Atlas (TCGA) in 2017, which had analyzed 228 CC samples, We may have had preferential access to samples from more advanced tumors/centers in the first partial analysis. In the same population (48%), we previously reported on a metagene, combining loss-of-function (LoF) in epigenetic regulators together with driver alterations in the *PIK3CA* pathway, which we revealed to be correlated with poor PFS (*p*-value = 0.02, log-rank test^8^. Subsequently, a multi-omics combined analysis was published on data from 89 patients for whom protein data (RPPA), complete clinical data, whole-exome sequencing, or analysis of a DNA panel of 608 genes alongside copy number variations (CNV) were available. A CoxBoost analysis detected the presence of a post-translational modification of β-catenin at Serine 552 (*β*-Cat-Ser552) as significantly associated with poor PFS, while another post-translational modification of the same protein was linked to good PFS^9^.

More recently, an independent pathology review of the complete patient set revealed that the detection of any sign of necrotic tissue debris in the baseline pathological sample was highly significantly associated with poor PFS^10^, and this holds true in the latest CoxBoost model. Since necrosis has thus been documented as a high-profile biomarker, and since it is easily assessable by pathologists, we examined the gene expression profiles of tumors with and without necrosis at diagnosis. Differentially expressed genes were detected, but none of these had prognostic value on their own.

For the present manuscript, we were able to analyze gene fusions and to highlight *FGFR3* fusions (5%) to be slightly more frequent in our cohort than previously reported in the literature (3%)^13^. The *FGFR3*-*TACC3* fusion gene has been well documented in muscle-invasive bladder cancer^14^ and cholangiocarcinoma^15^ as well as in CC^16^. The presence of *FGFR3* fusion genes confers sensitivity to anti-FGFR3 agents such as erdafitinib, which is currently being evaluated in bladder cancer. *BRCA2* alterations were observed in 2% of patients, suggesting potential clinical benefit in a few patients from PARP inhibitors. PARP inhibitors have been proposed to benefit patients identified with homologous repair deficiency (HRD) (4% in Bio-RAIDs), although this has been little documented in cervical cancer. In the presence of HRD, the use of PARP inhibitors alone or in conjunction with immune checkpoint inhibitors (ICIs), such as in the NCT04483544 trial^17^, is supported by clinical evidence in other cancers^18,19^. Anti-PD-L1 immunotherapy, either as monotherapy^5^ or in combination with chemotherapy^20^ is dependent on PD-L1 expression. The current immunohistochemical assessment by combined positive score (CPS) as a “predictive” biomarker of sensitivity to ICIs may be overrated. Tumor mutational burden (TMB) is a well-studied marker of sensitivity to ICIs in a variety of cancers. Pembrolizumab is approved for the treatment of metastatic tumors with TMB-high that have progressed following standard treatment^21^. The 11% TMB-high rate in our cohort of primarily locally advanced CC suggests that its assessment should be included in the routine work-up of CC patients. Microsatellite instability-high (MSI-high), another well-characterized predictive biomarker of ICI sensitivity documented in endometrial and metastatic colorectal tumors, was low in the present series (1%).

To identify prognostic expression biomarkers, a Cox proportional hazards regression model was used to identify genes with expression levels associated with progression-free survival (PFS). Among these, *TREM*1 (**t**riggering of **r**eceptor **e**xpressed on myeloid cells 1), a regulator of antitumor T cell immunity, was conserved in a separate volcanoplot based on the Cox regression survival analysis (Fig. 2C) and may be of clinical interest. Ajith A et al demonstrated that genetic or pharmacological silencing (using a small molecule inhibitor: VJDT) significantly delayed tumor growth in murine melanoma or fibrosarcoma models^22^.

The progression of cervical cancer is the result of a complex interplay of molecular mechanisms. We previously demonstrated that tumors with multiple genetic alterations were associated with increasingly poorer outcomes^8^. Of clinical interest, not previously described to our knowledge, was the detection of *TERT* (telomerase reverse transcriptase) promoter mutations in 13% of patients, while other clinically actionable alterations, such as *FGFR3* gene fusions (5%) and *BRCA2* alterations (4%), were less frequent. *TERT* was significantly associated with poor PFS in a CoxBoost model. Pathway-based analysis revealed a significant correlation between *TERT* promoter mutations and the senescence pathway, both being associated with poor PFS, consistent with the senescence pathway being driven by *TERT*. *TERT* is a catalytic subunit of telomerase, which is well documented to play a significant role in the initiation and progression of numerous cancer types^23^. Alterations of *TERT* have been reported in numerous cancer types, and oncogenic events appear to result from amplifications, promoter mutations, or single-nucleotide variants (SNVs)^24^. To our knowledge, the negative impact of *TERT* promoter mutations on prognosis had not been reported in the literature for CC. These results are of interest in the context of the development of reagents such as INO-5401, a *TERT*-encoding DNA vaccine composed of a modified *TERT* antigen alongside two additional cancer antigens, *WT1* and *PSMA*. This vaccine has shown promising results in glioblastoma^25^. Of high interest are also our findings on the clinical follow-up of these patients, who could be successfully surveyed by screening for HPV detection in the circulation^26^. Additionally, through a central pathological review, we identified the presence of tumor necrosis^10^ as a prognostic biomarker of high interest, due to its availability at diagnosis at no extra cost. When performing multi-omics analyses using a CoxBoost model approach, we demonstrated that the presence of necrosis at initial diagnosis, which can be identified through histopathological analysis of the tumor, was significantly correlated with poorer progression-free survival (PFS). This observation is relevant, as the impact of molecular alterations did not emerge as significant in our CoxBoost model, such as the presence of a *TERT* alteration, for instance. This result could be explained by the fact that in multi-omics models, the weight of molecular alterations is typically overshadowed by transcriptomic expression data. However, identifying necrosis in the surgical specimen is an inexpensive and easily obtainable piece of information that could help clinicians stratify patient risk. Our multivariable analyses confirmed that all two proposed biomarkers were independently associated with PFS after adjustment for established clinical covariates. This finding underscores the robustness of the associations. Based on this large prospectively collected CC dataset (n = 291), analyzed uniformly, the study reflects outcomes to standard therapy, but is limited by single-biopsy molecular analyses, which may not capture tumor heterogeneity. Further validation in larger and independent cohorts such as TCGA-CESC will be required to firmly establish the generalizability of our findings.

Our integrative multi-omics analyses generate a robust baseline genomic and transcriptomic resource in the under-explored field of cervical cancer (CC). Genomic alterations with potential clinical actionability were identified in over 50% of tumors, highlighting the relevance of next-generation sequencing for both precision oncology and translational research in CC. *TERT* alterations were associated with poorer progression-free survival (PFS), supporting their potential clinical significance. Transcriptomic analyses identified associations with specific genes, pathways, and baseline tumor necrosis.

## Methods

### Patients and samples

Consenting patients were enrolled in the EU-funded prospective CC Bio-RAIDs trial, which was conducted by the RAIDs Network (www.raids-fp7.eu). The clinical protocol has been registered at ClinicalTrials.gov (NCT02428842).

### All methods and study procedures were conducted in accordance with the Declaration of Helsinki

RAIDS was approved by a national research ethics committee (CPP Ile-de-France 1) and the French National Agency for the Safety of Medicines and Health Products (ANSM) on January 10th, 2013.

The study was conducted in 18 European institutions from seven countries, and tumor sampling procedures and quality control measures of samples and treatment have been previously reported^7,27^. All patients underwent pre-treatment mandatory frozen tumor biopsies, blood and serum sampling, as well as magnetic resonance imaging (MRI) examination. The option of undergoing a positron emission tomography (PET) computed tomography (CT) scan was available to half of the study population. A number of data sets were created to address specific questions based on shared patients and data types. A total of 419 patients had been included in the study, and annotated PFS clinical data are available for 377 patients. **Supplementary Fig. 1** provides a summary of the number of patients included into each analysis performed whenever contributive material was available.

### DNA/RNA extraction

Core samples of approximately 8 mm of tissue (approximately 30 mg of tissue) in each individual extraction were processed using the Allprep DNA/RNA Mini Kit, 50 samples (Qiagen ref. 80204) (if necessary, multiple cores were accumulated to reach the approximately 8 mm tissue length in each extraction). DNA extracts from frozen whole blood were performed using Chemagix DNA blood kit special 4 mL (Perkin-Elmer ref.CMG-1074).

For the present study, DNA was newly extracted from tumor samples obtained from the same patients, although not from the exact same biopsy. Sequencing was performed at a different facility using more recent-generation sequencing platforms than those used in the previously published analyses.

### Whole Exome Sequencing (WES)

1 µg of genomic DNA from each sample was subjected to shearing using Covaris. Illumina-compatible libraries were prepared according to the Roche KAPA Hyper Exome Prep protocol, consisting of repairing DNA ends and ligating Illumina barcoded adapters followed by PCR amplification. Libraries were pooled in equimolar condition before being hybridized on dedicated KAPA Exome Enrichment Probes (KAPA_HyperExome_hg38_capture_targets.bed). After selection using streptavidin beads and PCR amplification, enriched library pools were subjected to qPCR quantification using the KAPA library quantification kit Sequencing was

on the NovaSeq 6000 instrument from Illumina based on a 2*100 cycle mode (paired-end reads, 100 bases) using a S2 flow cell to obtain approximately 25 million clusters (50 million raw paired-end reads) per blood sample (30X target depth of coverage) and 75 million clusters (150 million raw paired-end reads) per tumor sample (100X target depth of coverage).

### Shallow Whole Genome Sequencing (sWGS)

KAPA pre-capture pools are used for low-coverage whole-genome sequencing of all samples. Sequencing was carried out on the NovaSeq 6000 instrument from Illumina based on a 2*100 cycle mode (paired-end reads, 100 bases) using a S1 flow cell to obtain around 4 million clusters (8 million raw paired-end reads) per sample.

### RNA sequencing

RNA sequencing libraries were prepared from 1 µg of total RNA using the Illumina TruSeq Stranded mRNA Library preparation kit, which allows for strand-specific sequencing. A first step of polyA selection using magnetic beads is done to focus sequencing on polyadenylated transcripts. After fragmentation, cDNA synthesis was performed, and resulting fragments were used for dA-tailing followed by ligation of TruSeq-indexed adapters. PCR amplification generated the final barcoded cDNA libraries. The libraries were equimolarly pooled and subjected to qPCR quantification using the KAPA library quantification kit (Roche). Sequencing was carried out on the NovaSeq 6000 instrument from Illumina based on a 2*100 cycle mode (paired-end reads, 100 bases) using a S1 flow cell to obtain around 30 million clusters (60 million raw paired-end reads) per sample.

### Bioinformatic DNA analysis

WES data have been analyzed using the Institut Curie Variant Calling Pipeline for whole-exome and whole-genome sequencing of Cancer data (VEGAN version 1.2.0, https://github.com/bioinfo-pf-curie/veganhttps://doi.org/10.5281/zenodo.12167638), as depicted in Supplementary Fig. 2. Briefly, the reads were mapped to the Human hg38 reference genome using the BWA-MEM algorithm. Only those reads that were uniquely mapped to the exome capture region with a minimum mapping quality of 20 were retained for subsequent analysis. Duplicates were removed using the Picard MarkDuplicates tool.

### Variant calling of somatic variants

Single nucleotide variants (SNVs) and insertions/deletions (indels) were identified and classified using Mutect2 (GATK v4.1.9.0). The annotation of somatic variants was performed with SnpEff (v.4.3t). Only coding variants that met the technical filters and satisfied the following criteria were reported:

1) Functional impact variants: inframe indel, frameshift indel, missense, stop gain, splicing, variant upstream TERT gene (5k bases)

2) At least a tumor read depth of ≥ 20 and a tumor frequency of ≥5%

3) Polymorphism with a frequency equal to or less than 0.1% in gnomAD (GATK hg38 AF only gnomAD v2)

### Copy number analysis

Copy number alterations were identified using Facets (v0.6.1) on matched normal-tumor sample pairs. Focal amplification of less than 10 Mb was defined as a gain of at least five copies from the estimated ploidy.

### Microsatellite instability (MSI)

*MSI status was* determined for a distribution of microsatellites using MSIsensor-pro (v.1.2.0). The MSI score cutoff value was 3%.

### Tumor mutational burden (TMB)

Tumor mutational burden (TMB) is a measurement of mutations carried by a tumor. TMB calculations were realized using the pyTMB script (v.1.4.0)^18^, developed by Institut Curie. Variants with low quality, non-coding, synonymous, and polymorphic characteristics (MAF < 0.001% in 1000 Genomes, gnomAD) were removed. Furthermore, variants with a depth of coverage <20, alternative allele coverage <2, and an allele frequency <0.05 were also excluded. TMB was subsequently divided into two groups: high (≥10 mutations/Mb) and low (<10 mutations/Mb).

### Mutational signatures

SigProfilerAssignment (v.0.0.30) was employed to extract known single-base substitutions (SBS) in accordance with COSMIC mutational signatures (v.3.3.1) on GRCh38. For each patient, only the most contributory mutational signature (≥20% of all mutations) was considered. Only the most contributive mutational signatures for each patient are reported in the Oncoprint annotation heatmap.

### HRD status

HRD status has been defined from shallow WGS using QDNAseq followed by shallowHRD (v.1.13, commit ef96eea, R - v.4.2.1). Following the shallowHRD recommendation, data were mapped with the BWA-MEM algorithm (v0.7.17-r1188) against the hg19 base reference. The number of large-scale genomic alterations (LGAs) was calculated on the autosomal and X chromosomes. The HRD status was assigned to patients with a minimum of 10 Mb LGAs, with at least 18, while the HRP status was assigned to patients with a minimum of 10 Mb LGAs, with a total of less than 18.

### Filtering variants and oncoprints

A dedicated strategy was employed to analyze somatic variants and copy number alterations, with consideration given to their functional impact on gene categories. These included oncogenes, tumor suppressor genes (TSGs), both, or unknown, based on the OncoKB database (v3.1, 2021 February). Focal amplifications (≤10 Mb) and activating variants were considered for oncogenes. These included pathogenic missense mutations known in the COSMIC, ICGC, or Cancer Hotspot databases, as well as inframe indels mutations known in the COSMIC, ICGC, or Cancer Hotspot databases. Homozygous deletions and inactivating variants were considered for TSG. These included: i) pathogenic missense mutations known in the COSMIC, ICGC, or Cancer Hotspot databases, ii) inframe indels mutations known in the COSMIC, ICGC, or Cancer Hotspot databases, and iii) truncating variants such as stop gains, frameshift indels, and variants disrupting splice sites. All aforementioned alterations were considered for both oncogenes and TSG, as well as for unknown genes. Oncoprints were visualized using the R (v.4.4.0) package Complex Heatmap (v.2.20.0), which demonstrated the most frequently altered genes (with a frequency of greater than 3%).

### Signaling pathways

All altered OncoKB genes according to our criteria above were grouped into 26 signaling pathways, with 257 genes that could not be classified.

### Druggable genes

Oncoprint of druggable genes showing the OncoKB actionable genes level 1, level 2, and level 3 (version 2024 March). OncoKB assigns levels of actionability based on evidence for the genomic alteration to serve as a biomarker either in that cancer type or in other cancer types.

### RNA analysis

The RNA-seq data were processed using the Institut Curie RNA-seq pipeline (v.4.1.0) available at https://github.com/bioinfo-pf-curie/RNA-seqhttps://doi.org/10.5281/zenodo.7837455. Following the removal of adapter sequences, the reads were aligned to the Human reference genome (hg38) using the STAR software (v2.7.6a). The gene expression matrix was generated with STAR using the --quanMode GeneCounts option. Coding genes with very low expression levels (TPM ≥ 1 in fewer than 5 samples) were excluded from the analysis. The count data were then normalized using the trimmed mean of M-values (TMM) method of the edgeR package. Differential expression analyses were performed using the R (v.4.2.1) package Limma-voom (v.3.54.2) and adjusted for multiple testing using the Benjamini-Hochberg method. Gene Set Enrichment Analysis (GSEA) was conducted using the R package fgsea (v.1.29.1) on MSigDB (v7.5.1) Hallmark gene sets using pre-ranked genes according to their assigned differential expression t-score. Benjamini-Hochberg method was applied to adjust the *p*-values.

Deconvolution of RNA-seq data was performed using CIBERSORT in absolute mode from the immunedeconv R package (v.2.1.0) to characterize stromal cell fractions. For each cellular type, a univariate Cox proportional hazard regression model was fitted using the square-root-transformed and scaled cellular fraction using the R package Survival (v3.5-8). Significance was assessed using the Wald test and adjusted for multiple testing using the Benjamini-Hochberg method.

### Fusion detection and validation

The RNA-fusion 1.1.0 pipeline from Institut Curie was applied for fusion calling. This involved the use of STAR-fusion, FusionCatcher, and Arriba to identify fusion genes (Supplementary Fig. 3).

The fusion transcripts of biological interest were identified manually, with the OncoKB database being used to select the most relevant and plausible fusion genes. The algorithm used to select the fusions of interest was as follows:

Firstly, the genes involved in the fusion were classified as either oncogenes or tumor suppressor genes. Oncogenes are genes involved in carcinogenesis through gain-of-function mechanisms, often encoding a protein involved in a signaling pathway necessary for cell survival. In contrast, tumor suppressor genes are genes involved in carcinogenesis through loss-of-function mechanisms, for example, encoding a protein ensuring genome integrity.

### Secondly, based on this classification, the following fusions were retained

In the case of fusions involving a tumor suppressor gene, so-called “frameshift” fusions were identified, which cause a reading frame shift and thus result in the loss of function. In contrast, fusions involving an oncogene were found to be in-frame fusions (without a reading frame shift), which preserve the functional domain of the oncogene.

### Statistical analysis

The endpoint of interest was progression-free survival (PFS), defined as the minimal time to relapse or death. A differential mutation analysis was conducted between the groups using the Chi-square test and Benjamini-Hochberg correction on genes from the Oncoprint database. The statistical analysis of mutational signatures was also performed using the aforementioned tests and corrections. Progression-free survival curves were estimated using the Kaplan-Meier method and were compared using log-rank tests using the R (v.4.4.0) package Survival (v3.5-8) and Survminer (v.0.4.9).

For each protein-coding gene, a univariate Cox proportional hazards regression model was fitted using the scaled normalized gene counts as the predictor using the R package Survival (v3.5-8). The scaled normalized gene counts correspond to the TMM log2 normalized counts followed by z-score scaling across samples. Significance was assessed using the log-rank test and adjusted for multiple testing using the Benjamini-Hochberg method.

Cox models with likelihood-based boosting were built to identify prognostic factors for progression-free survival using the R (v.4.3.0) package “CoxBoost”^17^). An integrative multi-omics analysis was performed, jointly modeling clinical variables together with genomic and transcriptomic data. Only patients with available clinical, genomic, and transcriptomic data were included in the analysis. Clinical variables were incorporated as unpenalized mandatory covariates, ensuring their systematic inclusion. These features were favored by setting them as an offset in the model. Molecular variables comprised gene-level DNA alterations and gene expression profiles derived from RNA-Seq and were subjected to boosting-based selection. A 10-fold cross-validation was used to determine the optimal number of boosting steps with respect to the partial log-likelihood.

## Supplementary information

Supplementary Figures_merged.

Supplementary Data.

## Data Availability

The original files and raw next-generation sequencing data generated in this study have been deposited in the EGA database under accession code EGAS50000001012. Data on EGA are under controlled access. Sequencing data will be made available upon request through EGA, and additional clinical information can be made available upon institutional approval. Requests should be addressed to J. Guerin and N. Servant (julien.guerin [@curie.fr](mailto:nicolas.servant@curie.fr) and nicolas.servant@curie.fr). The estimated timeframe for access to be granted is 2 months, and the duration will be determined according to the request needs. All relevant clinical trial data used in this study are accessible in the Source Data files and are deidentified. Source data are provided with this paper.

## Code availability

Source codes are available in https://github.com/bioinfo-pf-curie/RAIDS.

## References

[1] Singh, D. et al. Global estimates of incidence and mortality of cervical cancer in 2020: a baseline analysis of the WHO Global Cervical Cancer Elimination Initiative. *Lancet Glob Health* **11**, e197–e206 (2023). 10.1016/S2214-109X(22)00501-0 PMC9848409 36528031

[2] Cibula, D. et al. ESGO/ESTRO/ESP Guidelines for the management of patients with cervical cancer – Update 2023*. *Int J. Gynecol. Cancer* **33**, 649–666 (2023). 10.1136/ijgc-2023-004429 PMC10176411 37127326

[3] Chao, X. et al. Selection of Treatment Regimens For Recurrent Cervical Cancer. *Front Oncol.* **2**, 618485 (2021). 10.3389/fonc.2021.618485 PMC7884815 33604304

[4] Li, J. et al. Cervical cancer prognosis and related risk factors for patients with cervical cancer: a long-term retrospective cohort study. *Sci Rep.* **17**, 13994 (2022). 10.1038/s41598-022-17733-8 PMC9385852 35978078

[5] Tewari, K. S. et al. Survival with Cemiplimab in recurrent cervical cancer. *N. Engl. J. Med* **386**, 544–555 (2022). 10.1056/NEJMoa2112187 35139273

[6] Tewari, K. S. et al. Bevacizumab for advanced cervical cancer: final overall survival and adverse event analysis of a randomised, controlled, open-label, phase 3 trial. *Gynecologic Oncology Group* **240**, 1654–1663 (2017). 10.1016/S0140-6736(17)31607-0 PMC5714293 28756902

[7] Ngo, C. et al. From prospective biobanking to precision medicine: BIO-RAIDs – an EU study protocol in cervical cancer. *BMC Cancer* **15**, 842 (2015). 10.1186/s12885-015-1801-0 PMC4632364 26531748

[8] Scholl, S. et al. Clinical and genetic landscape of treatment naive cervical cancer: Alterations in PIK3CA and in epigenetic modulators associated with sub-optimal outcome. *EBioMedicine* **43**, 253–260 (2019). 10.1016/j.ebiom.2019.03.069 PMC6562019 30952619

[9] Scholl, S. M. et al. Genetic markers and phosphoprotein forms of beta-catenin pβ-Cat552 and pβ-Cat675 are prognostic biomarkers of cervical cancer. *EBioMedicine.* **61**, 103049 (2020). 10.1016/j.ebiom.2020.103049 PMC7581879 33096476

[10] Chouchane-Mlik, O. et al. Systematic assessment of tumor necrosis at baseline in cervical cancer - An independent factor associated with poor outcome. *Hum. Pathol.* **143**, 62–70 (2024). 10.1016/j.humpath.2023.12.003 38135059

[11] Chakravarty, D. et al. OncoKB: A Precision Oncology Knowledge Base. *JCO Precis Oncol.* 2017:PO.17.00011 (2017). 10.1200/PO.17.00011 PMC5586540 28890946

[12] Newman, A. M. et al. Robust enumeration of cell subsets from tissue expression profiles. *Nat. Methods* **12**, 453–457 (2015). 10.1038/nmeth.3337 PMC4739640 25822800

[13] Integrated genomic and molecular characterization of cervical cancer. *Nature*. **543**, 378–384 (2017). 10.1038/nature21386 PMC5354998 28112728

[14] Robertson A. G. et al. Comprehensive molecular characterization of muscle-invasive bladder cancer. *Cell* 174:1033 (2018). 10.1016/j.cell.2018.07.036 PMC6297116 30096301

[15] Jain, A. et al. Cholangiocarcinoma with FGFR genetic aberrations: a unique clinical phenotype. *JCO Precis Oncol.* 20181‑12. 10.1200/PO.17.00080 35135097

[16] Carneiro, B. A. et al. FGFR3–TACC3: A novel gene fusion in cervical cancer. *Gynecol. Oncol. Rep.* **13**, 53–56 (2015). 10.1016/j.gore.2015.06.005 PMC4563584 26425723

[17] Diaz, J. Immunotherapy in combination with PARP inhibition in advanced cervical cancer patients functionally competent or deficient for the Fanconi Anemia Repair Pathway. clinicaltrials.gov; 2024 [cité 1 janv 2024]. Report No.: NCT04483544. Disponible sur: https://clinicaltrials.gov/study/NCT04483544.

[18] Wallace, N. A. et al. Catching HPV in the Homologous Recombination Cookie Jar. *Trends Microbiol* **28**, 191–201 (2020). 10.1016/j.tim.2019.10.008 31744663

[19] Hussain, S. S., Lundine, D., Leeman, J. E., Higginson, D. S. Genomic signatures in HPV-associated tumors. *Viruses*. **5**, (1998). 10.3390/v13101998 PMC8537705 34696429

[20] Monk, B. J. et al. First-Line Pembrolizumab + Chemotherapy Versus Placebo + Chemotherapy for persistent, recurrent, or metastatic cervical cancer: final overall survival results of KEYNOTE-826. *J. Clin. Oncol.* **41**, 5505–5511 (2023). 10.1200/JCO.23.00914 37910822

[21] Research, C for DE and. FDA approves pembrolizumab for adults and children with TMB-H solid tumors. *FDA* [Internet]. 2020 [cité 12 juin 2024]; Disponible sur: https://www.fda.gov/drugs/drug-approvals-and-databases/fda-approves-pembrolizumab-adults-and-children-tmb-h-solid-tumors.

[22] Ajith, A. et al. Targeting TREM1 augments antitumor T cell immunity by inhibiting myeloid-derived suppressor cells and restraining anti-PD-1 resistance. *J. Clin. Invest* **133**, e167951 (2023). 10.1172/JCI167951 PMC10617775 37651197

[23] Daniel, M., Peek, G. W. & Tollefsbol, T. O. Regulation of the human catalytic subunit of telomerase (hTERT). *Gene* **498**, 135–146 (2012). 10.1016/j.gene.2012.01.095 PMC3312932 22381618

[24] Dratwa, M., Wysoczańska, B., Łacina, P., Kubik, T., Bogunia-Kubik, K. TERT—Regulation and Roles in Cancer Formation. *Front Immunol*. 2020 [cité 23 mai 2024];11. Disponible sur: https://www.frontiersin.org/journals/immunology/articles/10.3389/fimmu.2020.589929/full. 10.3389/fimmu.2020.589929 PMC7717964 33329574

[25] Reardon, D. A. et al. Intramuscular (IM) INO-5401 + INO-9012 with electroporation (EP) in combination with cemiplimab (REGN2810) in newly diagnosed glioblastoma. *J. Clin. Oncol.* **40**, 2004–2004 (2022).

[26] Jeannot, E. et al. Circulating HPV DNA as a marker for early detection of relapse in patients with cervical cancer. *Clin. Cancer Res. J. Am. Assoc. Cancer Res. 1* **27**, 5869–5877 (2021). 10.1158/1078-0432.CCR-21-0625 PMC9401545 34210686

[27] Samuels, S. et al. Precision medicine in cancer: challenges and recommendations from an EU-funded cervical cancer biobanking study. *Br. J. Cancer* **115**, 1575–1583 (2016). 10.1038/bjc.2016.340 PMC5155353 27875525

