# Supplementary material for: Integrative genomic and transcriptomic profiles from a prospective cervical cancer study
Source: NPJ Precis Oncol. 2026 Jul 22;10:437. doi: 10.1038/s41698-026-01616-6 (PMC13754857; doi:10.1038/s41698-026-01616-6)
Supplement: Supplementary file 1 [file 41698_2026_1616_MOESM1_ESM.pdf]

A

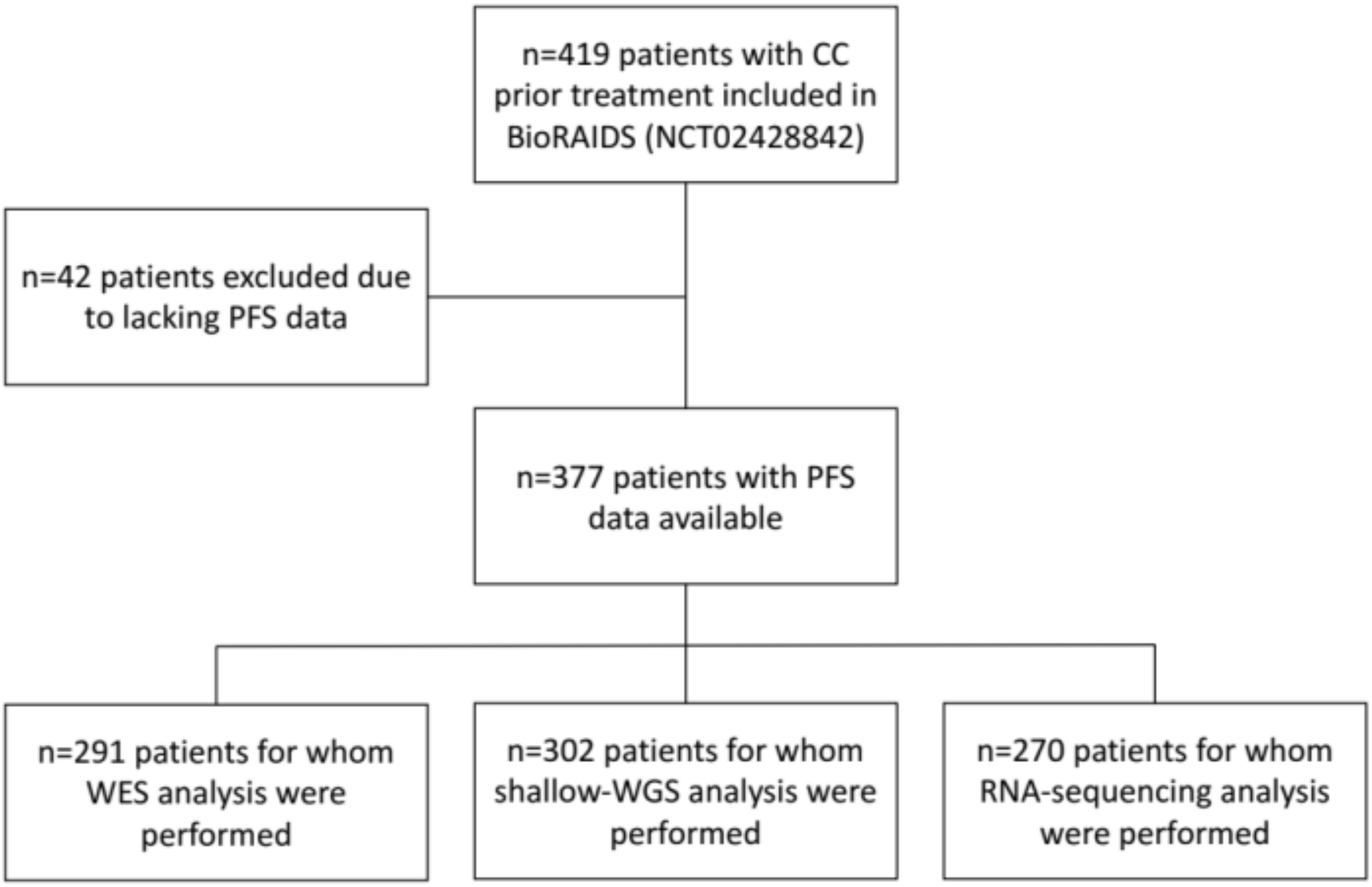

Supplementary Figure 1A. Study design and patient cohort inclusion.
This flow chart illustrates the inclusion process of patients with cervical cancer (CC) from the Bio-RAIDs. (PFS: Progression-free survival; WES: Whole Exome Sequencing; sWGS: shallow Whole Genome sequencing; RNA-Seq: RNA sequencing).

B

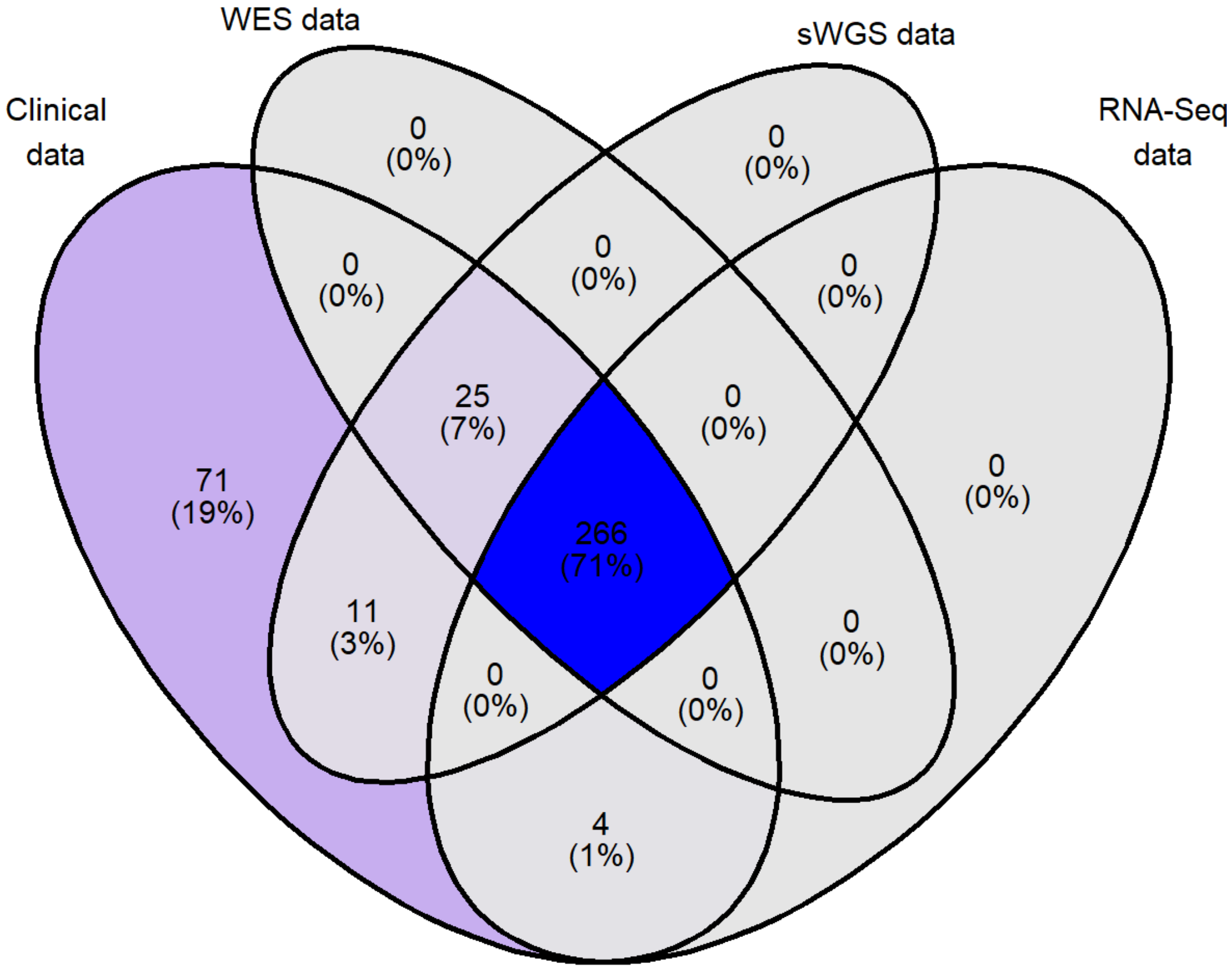

Supplementary Figure 1B. Venn diagram illustrating the number of patients for the different combinations of omics types and clinical data used in Bio-RAIDsS. (WES: Whole Exome Sequencing; sWGS: shallow Whole Genome sequencing; RNA-Seq: RNA sequencing).

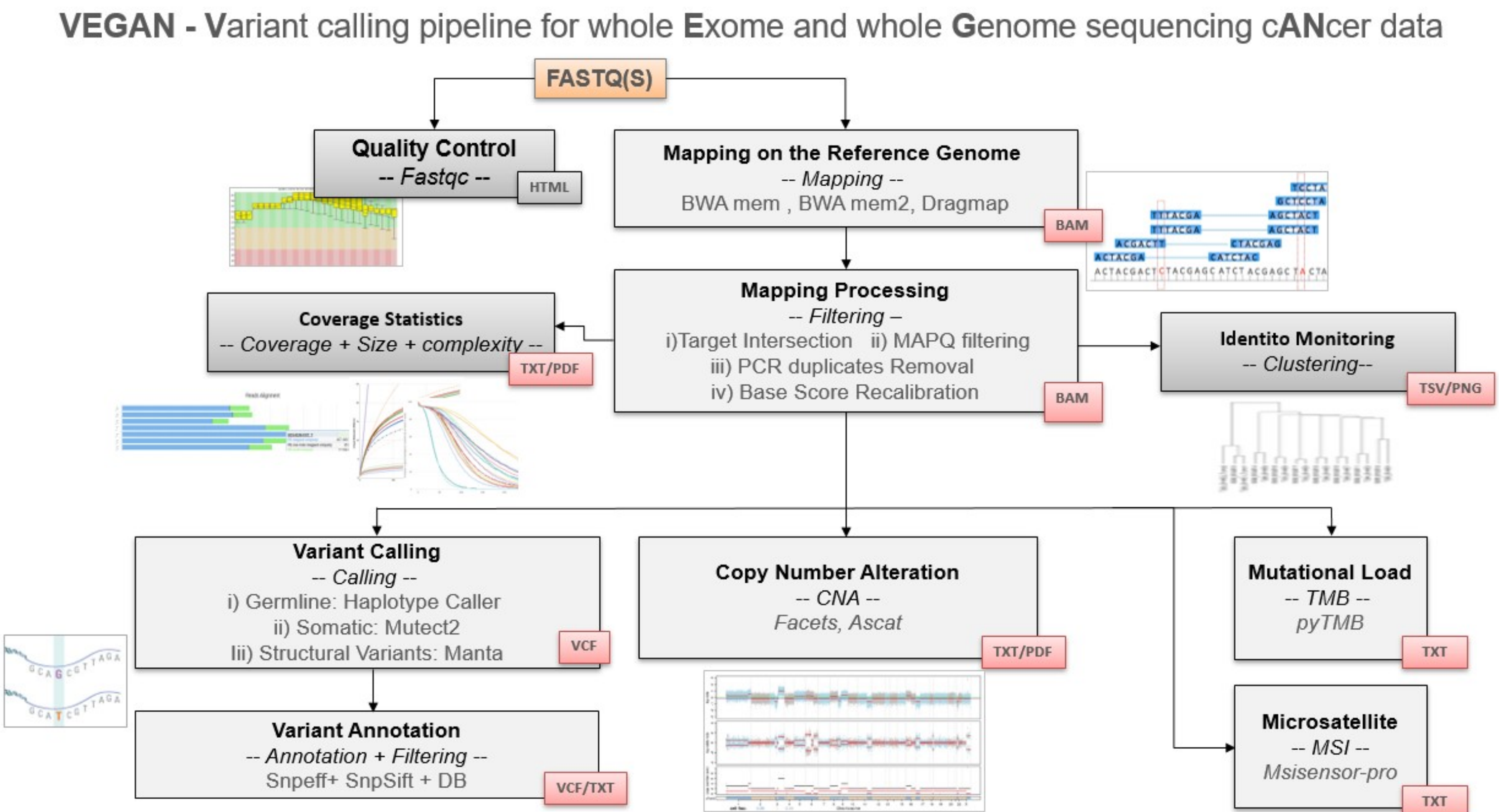

Supplementary Figure 2. Curie's bioinformatic pipeline (VEGAN)

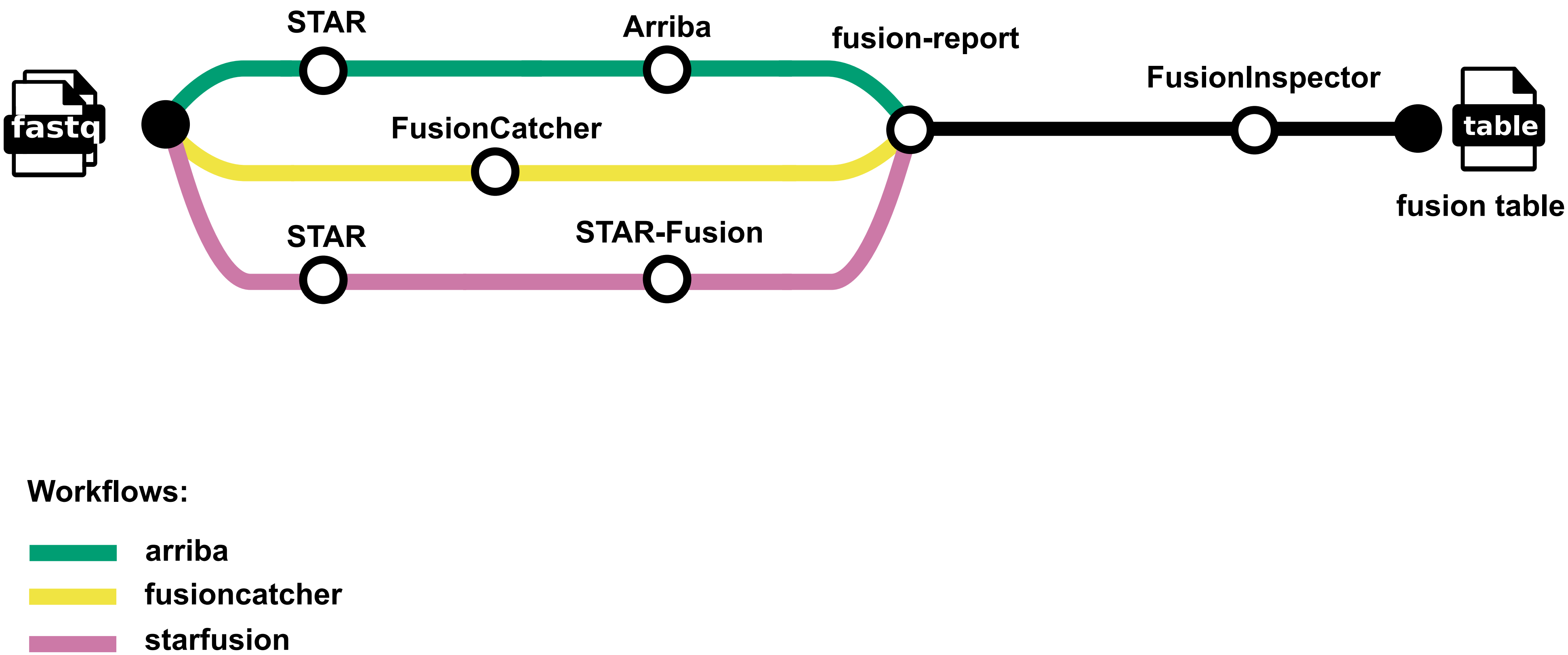

Supplementary Figure 3. Various tools comprising the RNAfusion 1.1.0 pipeline at the Curie Institute, used for the analysis of fusion transcripts.

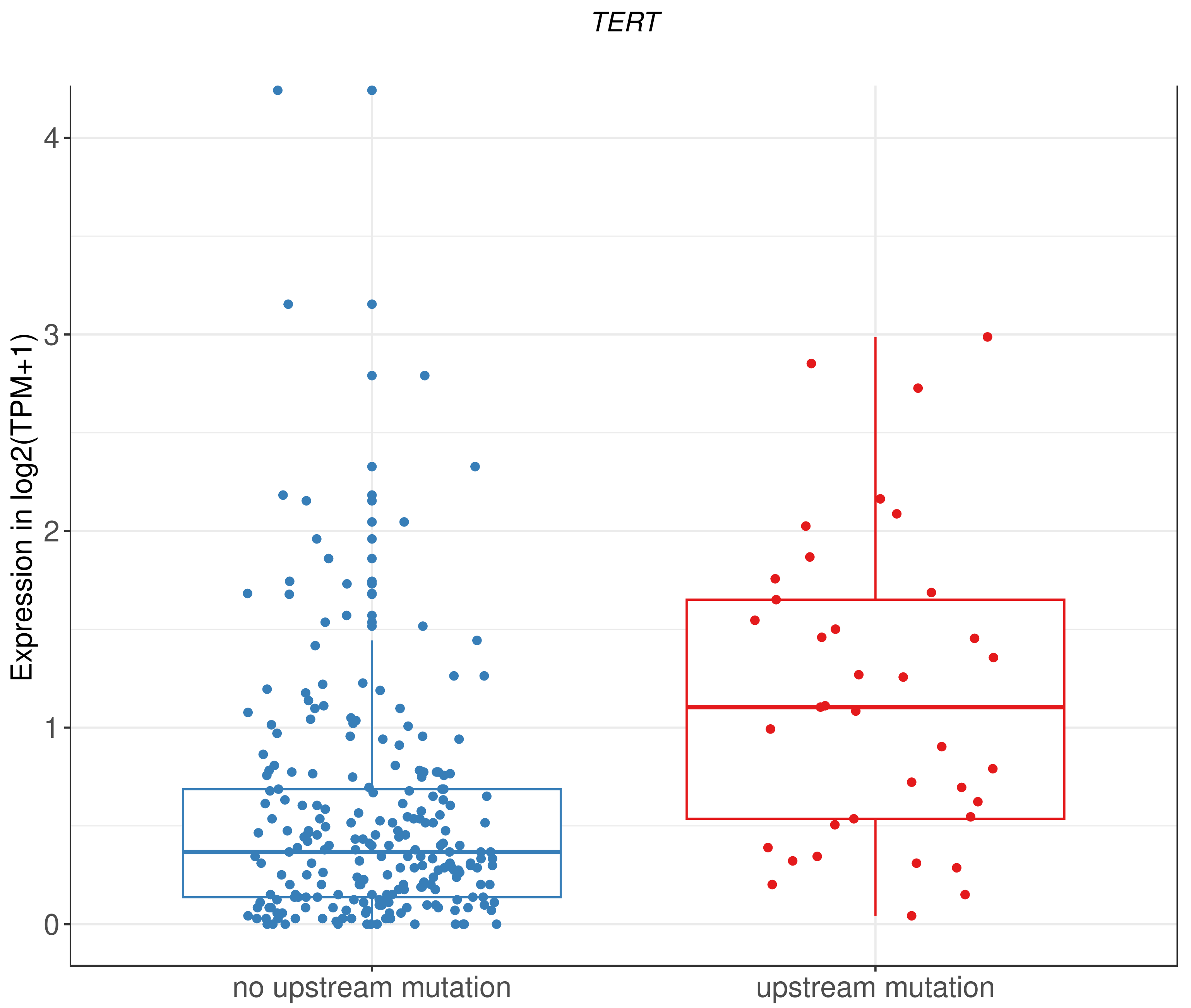

Supplementary Figure 4. Boxplot showing level expression (represented by a logarithmic scale on the left) of TERT according to the presence (mutated) or absence (wild-type) of an upstream mutation

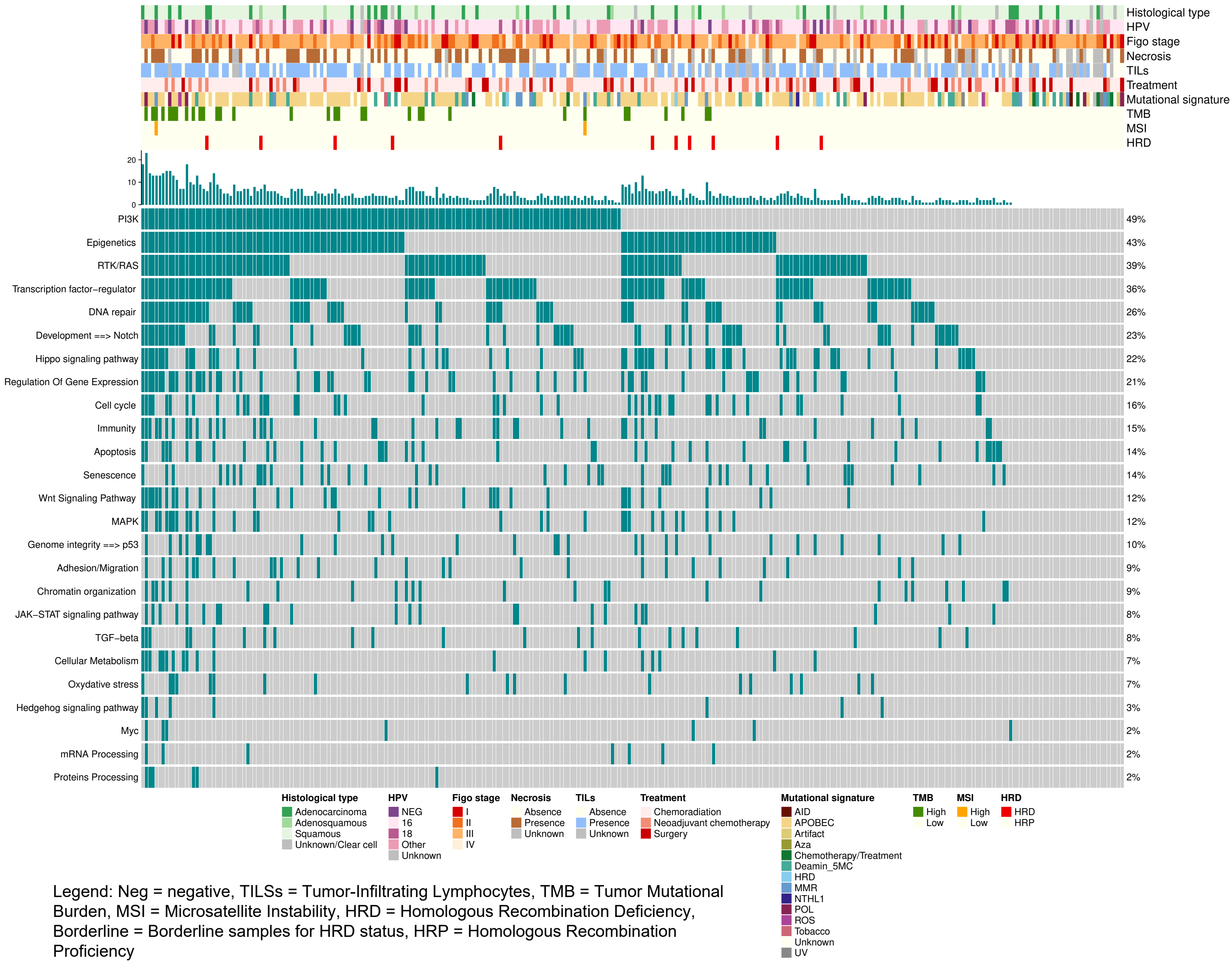

Supplementary Figure 5. Oncoprints presenting an overview of the most frequently altered (≥2%) signaling pathways

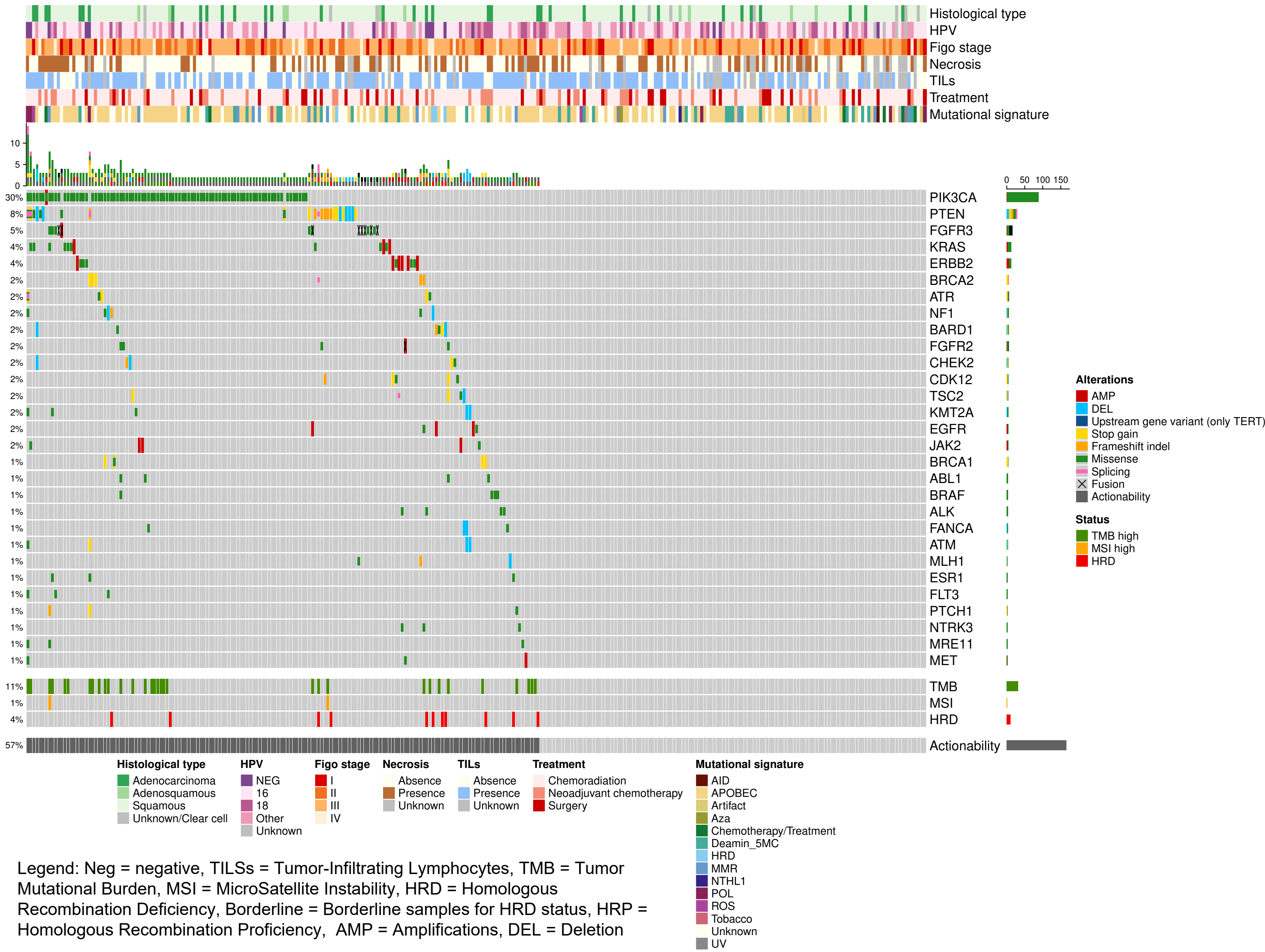

Legend: Neg = negative, TILSs = Tumor-Infiltrating Lymphocytes, TMB = Tumor Mutational Burden, MSI = MicroSatellite Instability, HRD = Homologous Recombination Deficiency, Borderline = Borderline samples for HRD status, HRP = Homologous Recombination Proficiency, AMP = Amplifications, DEL = Deletion

Supplementary Figure 6. Oncoprints presenting an overview of the most frequently altered (≥3%) oncogenes and tumor suppressor genes with clinical actionability, according to OncoKB's classification

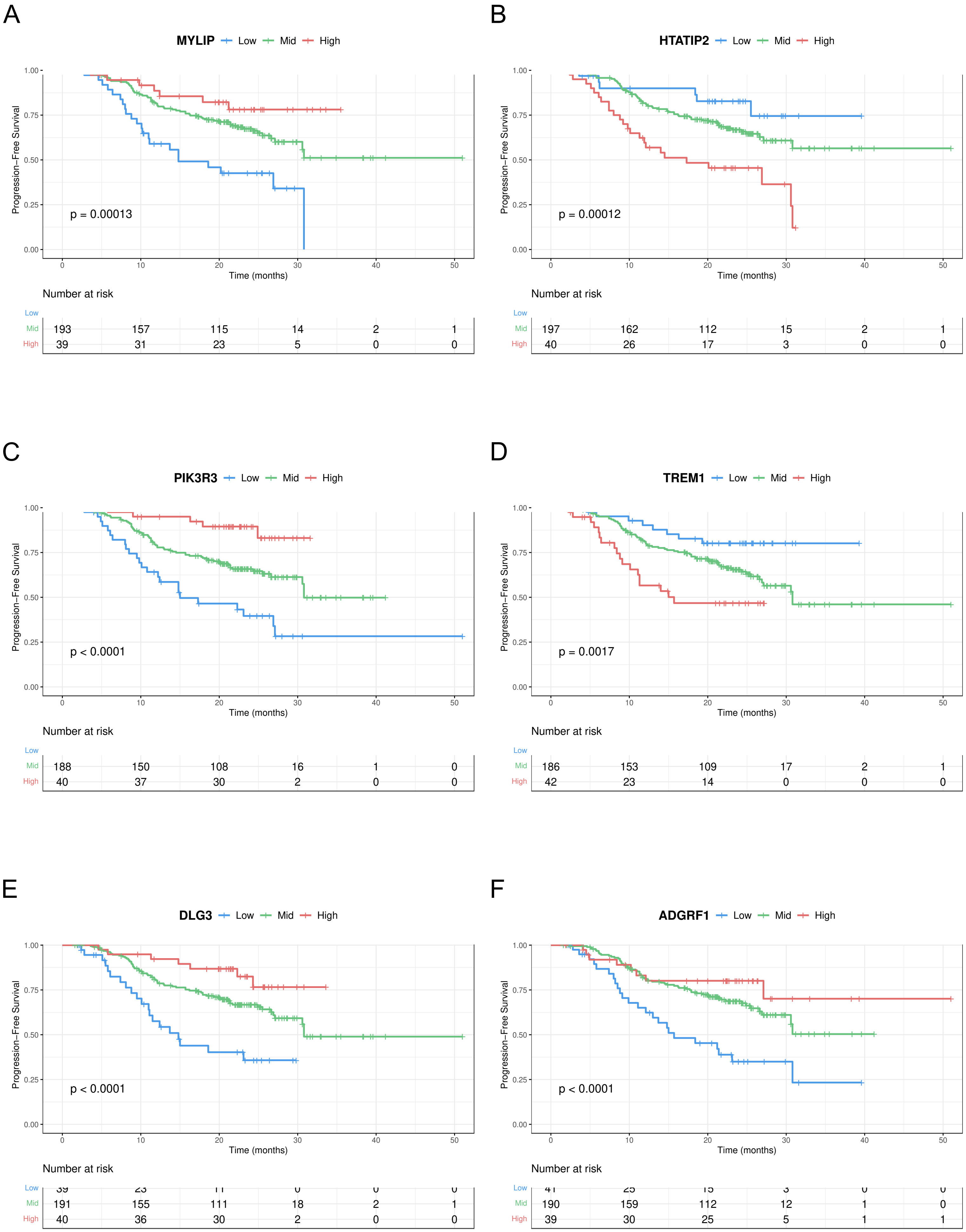

A
MYLIP Low Mid High
p = 0.00013
Progression−Free Survival
Time (months)
Number at risk
Mid 193 157 115 14 2 1
High 39 31 23 5 0 0
B
HTATIP2 Low Mid High
p = 0.00012
Progression−Free Survival
Time (months)
Number at risk
Mid 197 162 112 15 2 1
High 40 26 17 3 0 0
C
PIK3R3 Low Mid High
p < 0.0001
Progression−Free Survival
Time (months)
Number at risk
Mid 188 150 108 16 1 0
High 40 37 30 2 0 0
D
TREM1 Low Mid High
p = 0.0017
Progression−Free Survival
Time (months)
Number at risk
Mid 186 153 109 17 2 1
High 42 23 14 0 0 0
E
DLG3 Low Mid High
p < 0.0001
Progression−Free Survival
Time (months)
Number at risk
Mid 191 155 111 18 2 1
High 40 36 30 2 0 0
F
ADGRF1 Low Mid High
p < 0.0001
Progression−Free Survival
Time (months)
Number at risk
Mid 190 159 112 12 1 0
High 39 30 25 5 1 1

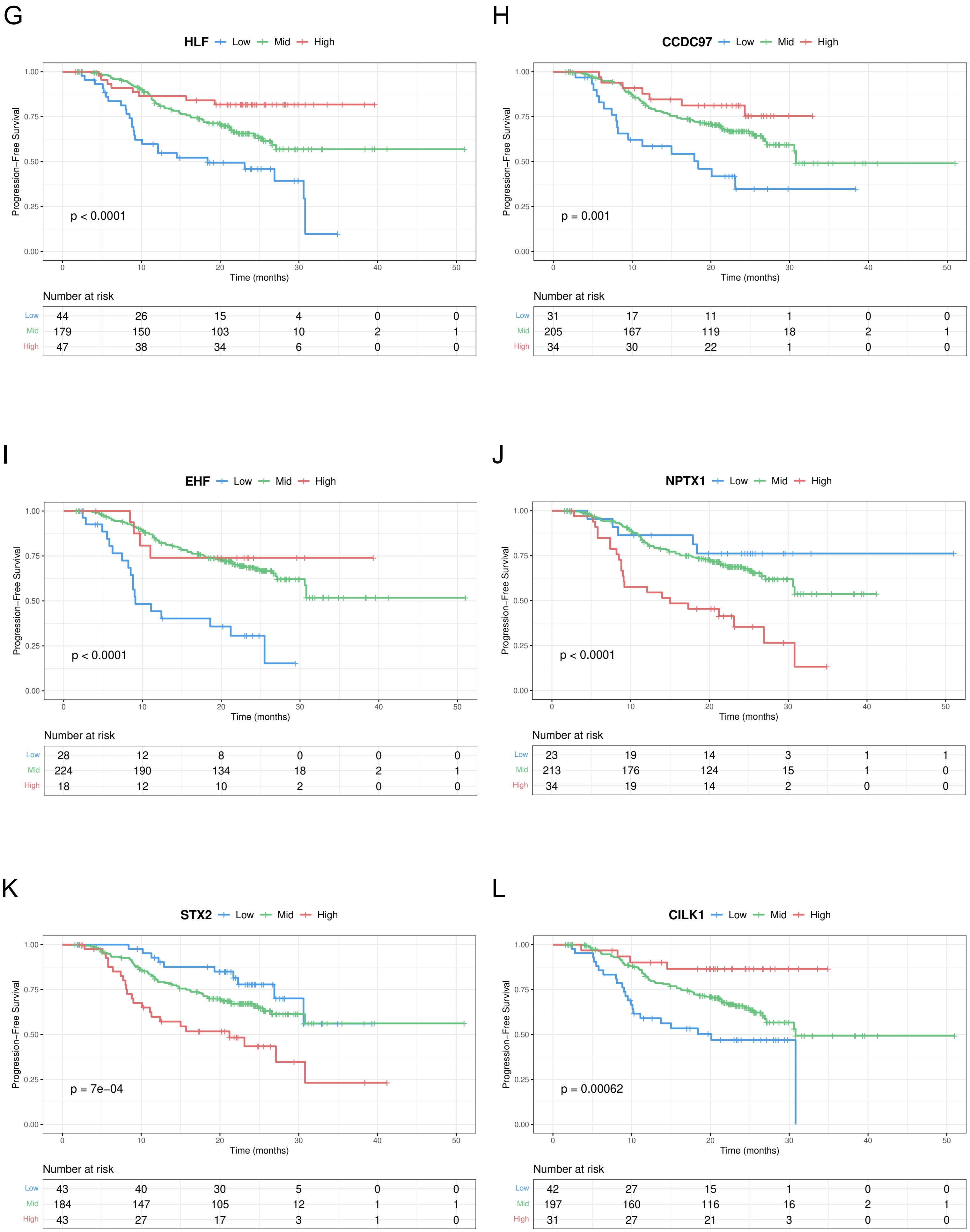
G
HLF Low Mid High
Progression−Free Survival
1.00
0.75
0.50
0.25
0.00
p < 0.0001
0 10 20 30 40 50
Time (months)
Number at risk
Low 44 26 15 4 0 0
Mid 179 150 103 10 2 1
High 47 38 34 6 0 0
H
CCDC97 Low Mid High
Progression−Free Survival
p = 0.001
Time (months)
Number at risk
Low 31 17 11 1 0 0
Mid 205 167 119 18 2 1
High 34 30 22 1 0 0
I
EHF Low Mid High
Progression−Free Survival
p < 0.0001
Time (months)
Number at risk
Low 28 12 8 0 0 0
Mid 224 190 134 18 2 1
High 18 12 10 2 0 0
J
NPTX1 Low Mid High
Progression−Free Survival
p < 0.0001
Time (months)
Number at risk
Low 23 19 14 3 1 1
Mid 213 176 124 15 1 0
High 34 19 14 2 0 0
K
STX2 Low Mid High
Progression−Free Survival
p = 7e−04
Time (months)
Number at risk
Low 43 40 30 5 0 0
Mid 184 147 105 12 1 1
High 43 27 17 3 1 0
L
CILK1 Low Mid High
Progression−Free Survival
p = 0.00062
Time (months)
Number at risk
Low 42 27 15 1 0 0
Mid 197 160 116 16 2 1
High 31 27 21 3 0 0

M

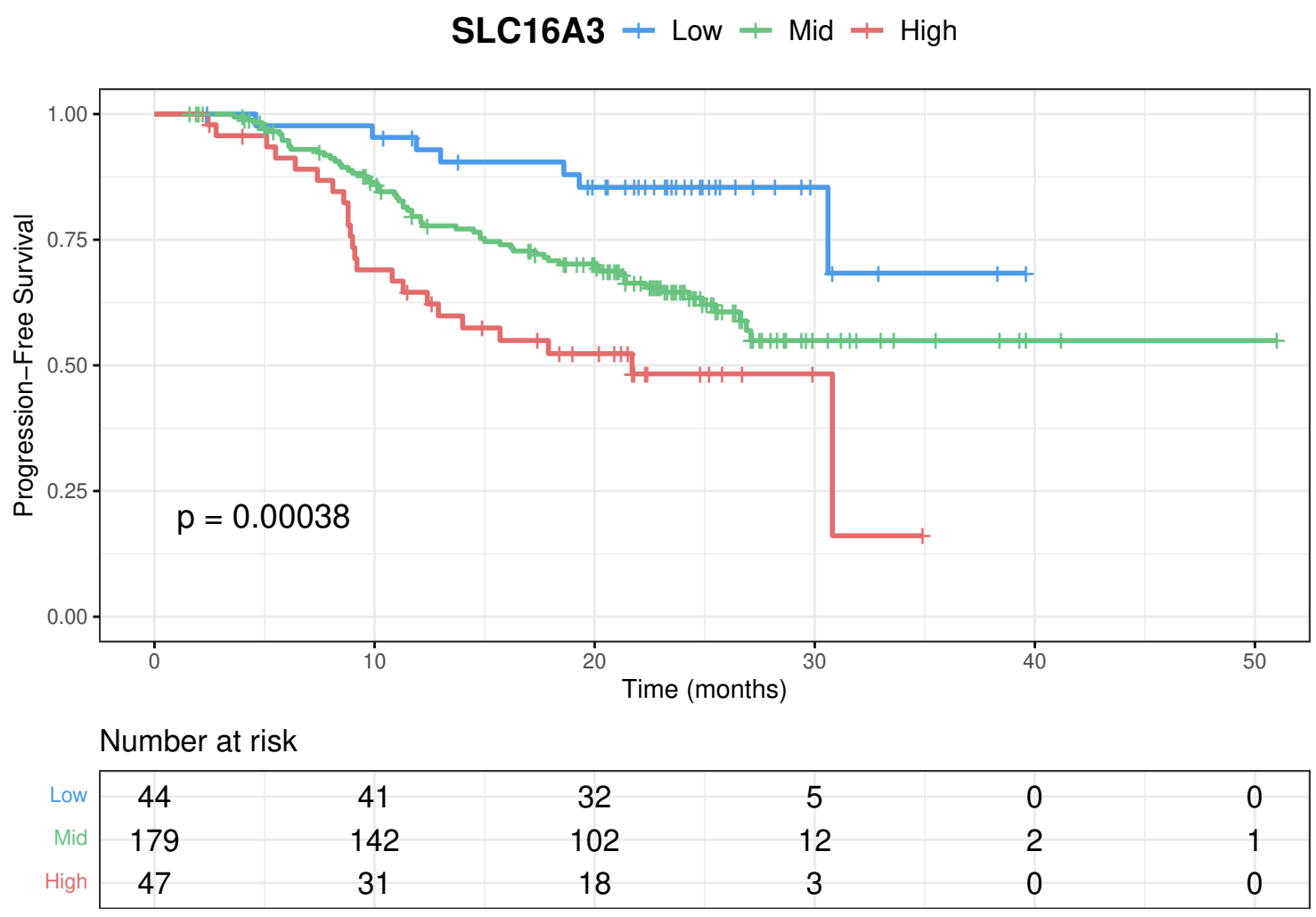

N

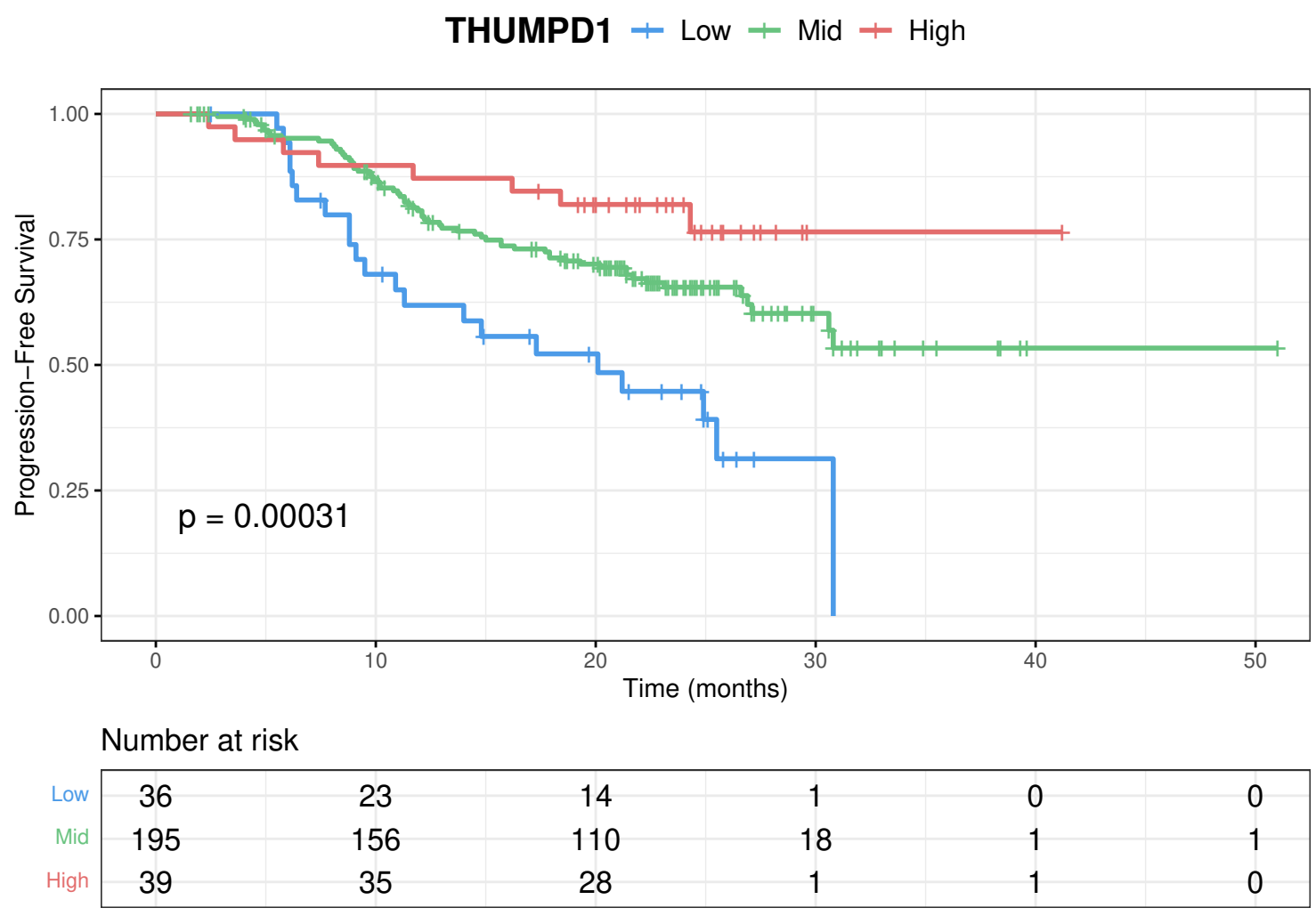

O

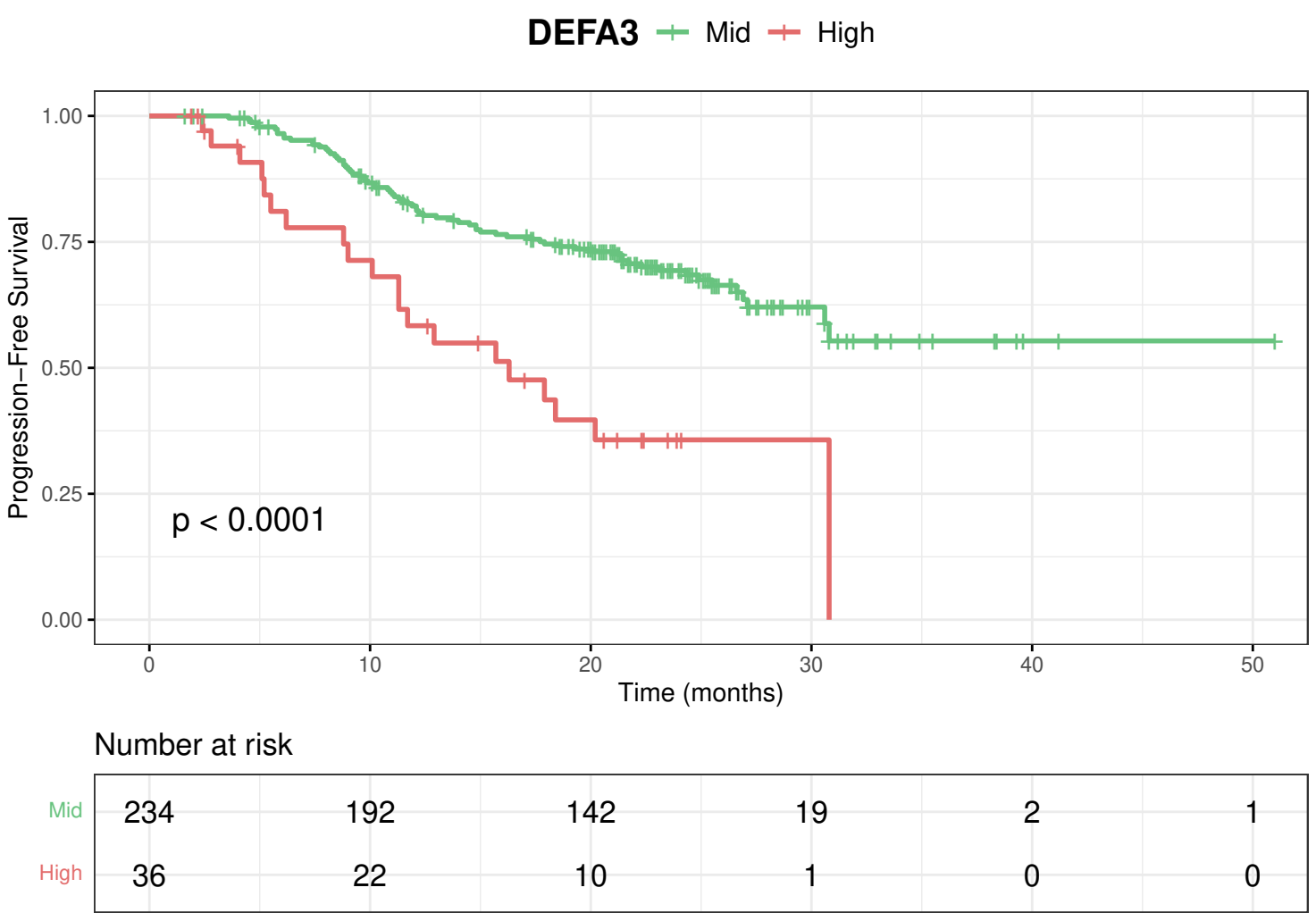

P

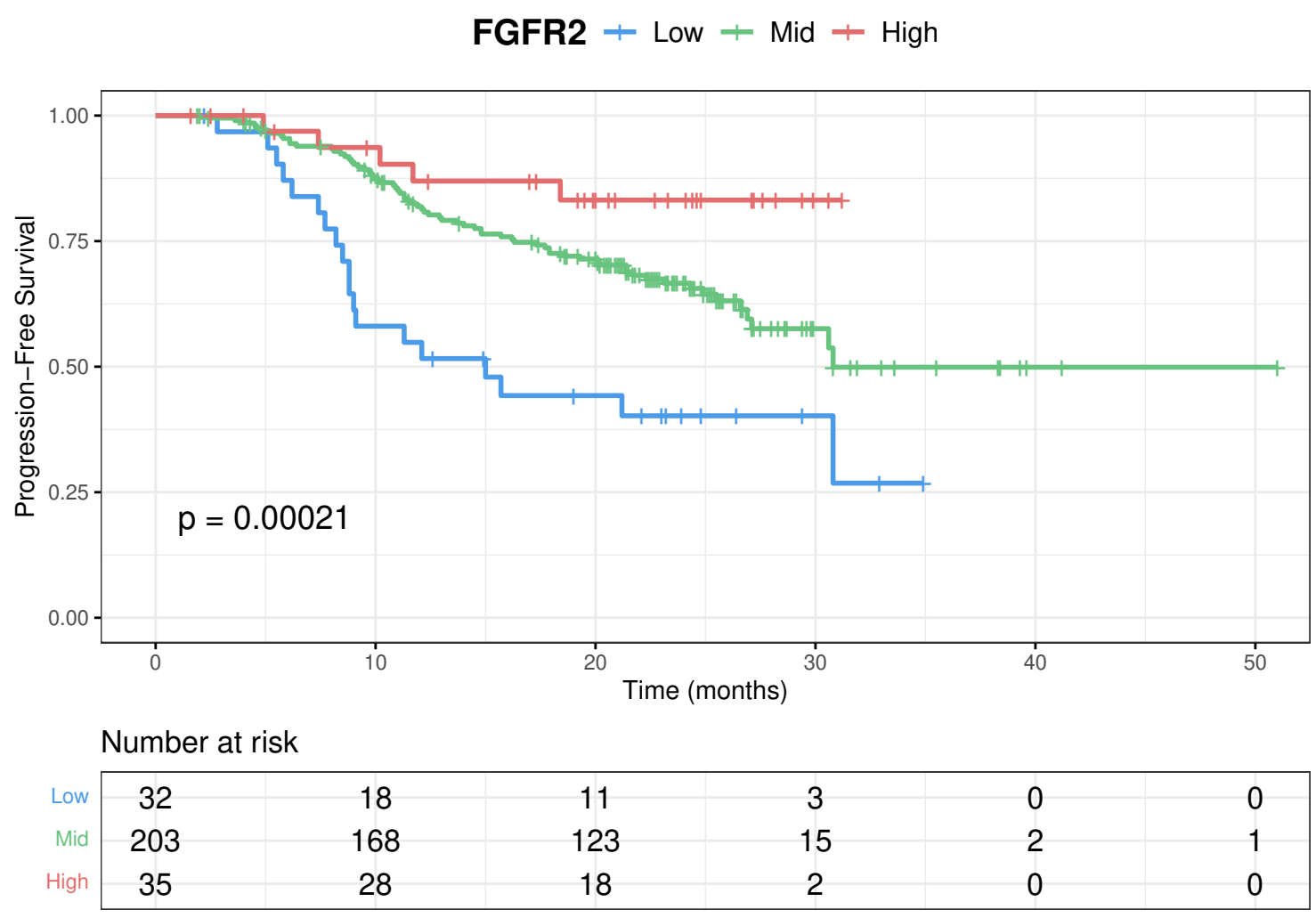

Q

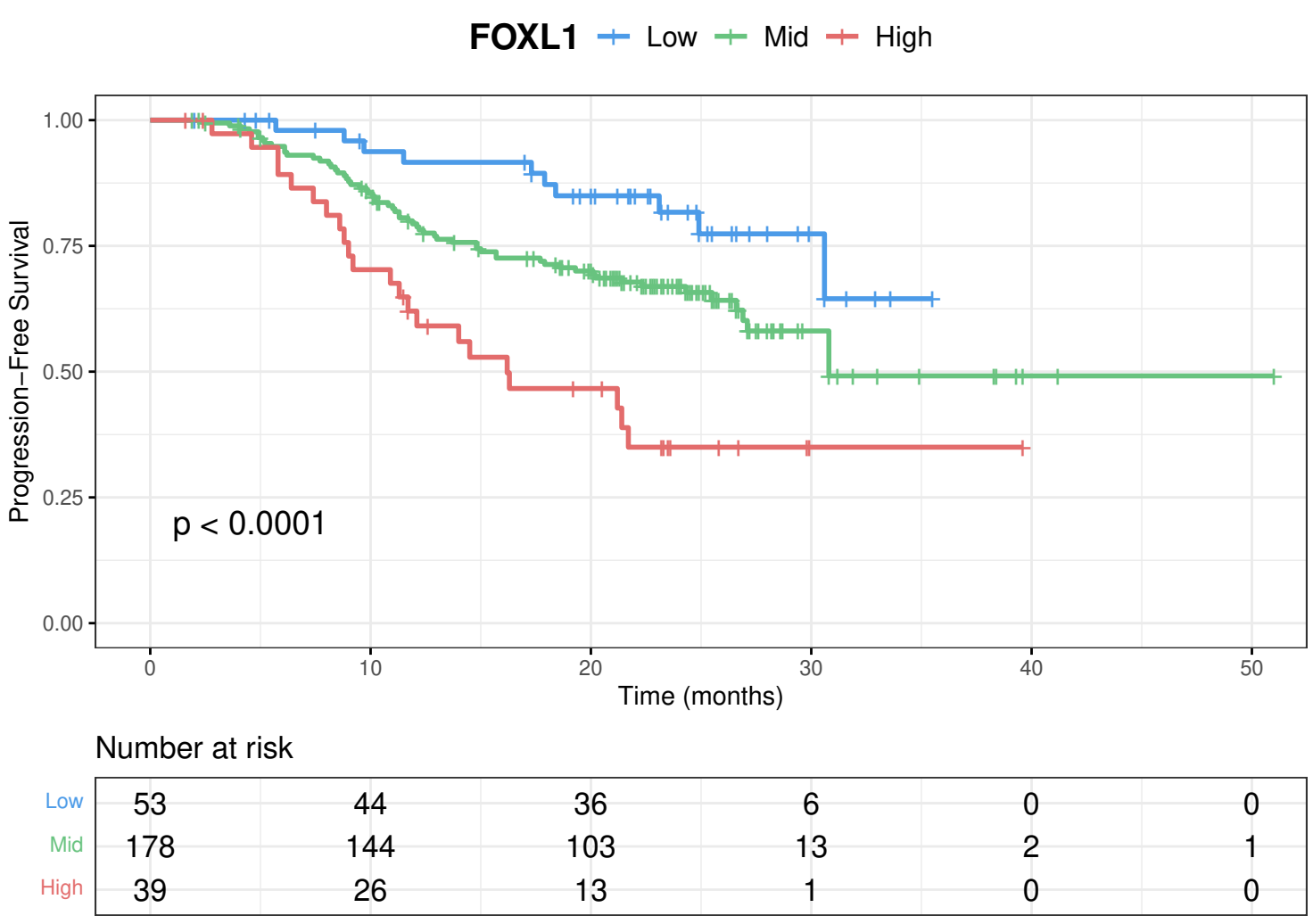

Supplementary Figure 7. Kaplan-Meier curves show progression-free survival according to scaled, normalized expression of the indicated genes, categorized as high (>1), mid (-1 to 1), and low (<-1). Group differences were assessed using the Log-rank test.
Subfigures: A-Q correspond to genes MYLIP, HTATIP2, PIK3R3, TREM1, DLG3, ADGRF1, HLF, CCDC97, EHF, NPTX1, STX2, CILK1, SLC16A3, THUMPD1, DEFA3, FGFR2, FOXL1, respectively

Univariate Cox Regression Results for CIBERSORTabs Cellular Fraction				
Model	HazardRatio	CI.95	p-value	p.adj.BH
T cell regulatory	0.64457	[0.51378;0.80866]	0.00014741	0.00324
T cell CD4 + naive	1.22185	[1.09475;1.36370]	0.00034991	0.00385
T cell follicular helper	0.70912	[0.57685;0.87171]	0.0011001	0.00807
B cell naive	0.73344	[0.58461;0.92017]	0.0073864	0.04063
Macrophage M1	0.77782	[0.63336;0.95524]	0.016538	0.07277
T cell CD8 +	0.78343	[0.62703;0.97885]	0.031704	0.09964
Myeloid dendritic cell resting	0.78843	[0.63850;0.97356]	0.027179	0.09964
Neutrophil	1.22	[1.01;1.47]	0.0389	0.10697
Myeloid dendritic cell activated	0.86381	[0.70324;1.06105]	0.16294	0.32588
Mast cell activated	0.86037	[0.69733;1.06153]	0.16064	0.32588
Mast cell resting	1.14363	[0.95573;1.36848]	0.14277	0.32588
Macrophage M2	0.87262	[0.70916;1.07376]	0.19793	0.36287
B cell plasma	0.87954	[0.70662;1.09476]	0.25042	0.42379
Monocyte	1.11097	[0.91146;1.35416]	0.29741	0.46736
T cell CD4 + memory activated	0.90559	[0.73626;1.11387]	0.34777	0.47818
NK cell activated	1.10160	[0.90754;1.33716]	0.32772	0.47818
B cell memory	0.93444	[0.76192;1.14604]	0.515	0.62944
Macrophage M0	0.93245	[0.75736;1.14801]	0.5098	0.62944
T cell CD4 + memory resting	0.95681	[0.78748;1.16256]	0.65684	0.76055
T cell gamma delta	1.03579	[0.84464;1.27020]	0.73549	0.80904
NK cell resting	0.99149	[0.80768;1.21712]	0.93487	0.93487
Eosinophil	1.01013	[0.82899;1.23085]	0.92039	0.93487

Supplementary Figure Table 1. Univariate Cox regression of each immune cell fraction estimated by CIBERSORT (absolute mode) for progression-free survival. Wald test p-values were adjusted using the Benjamini-Hochberg method

A

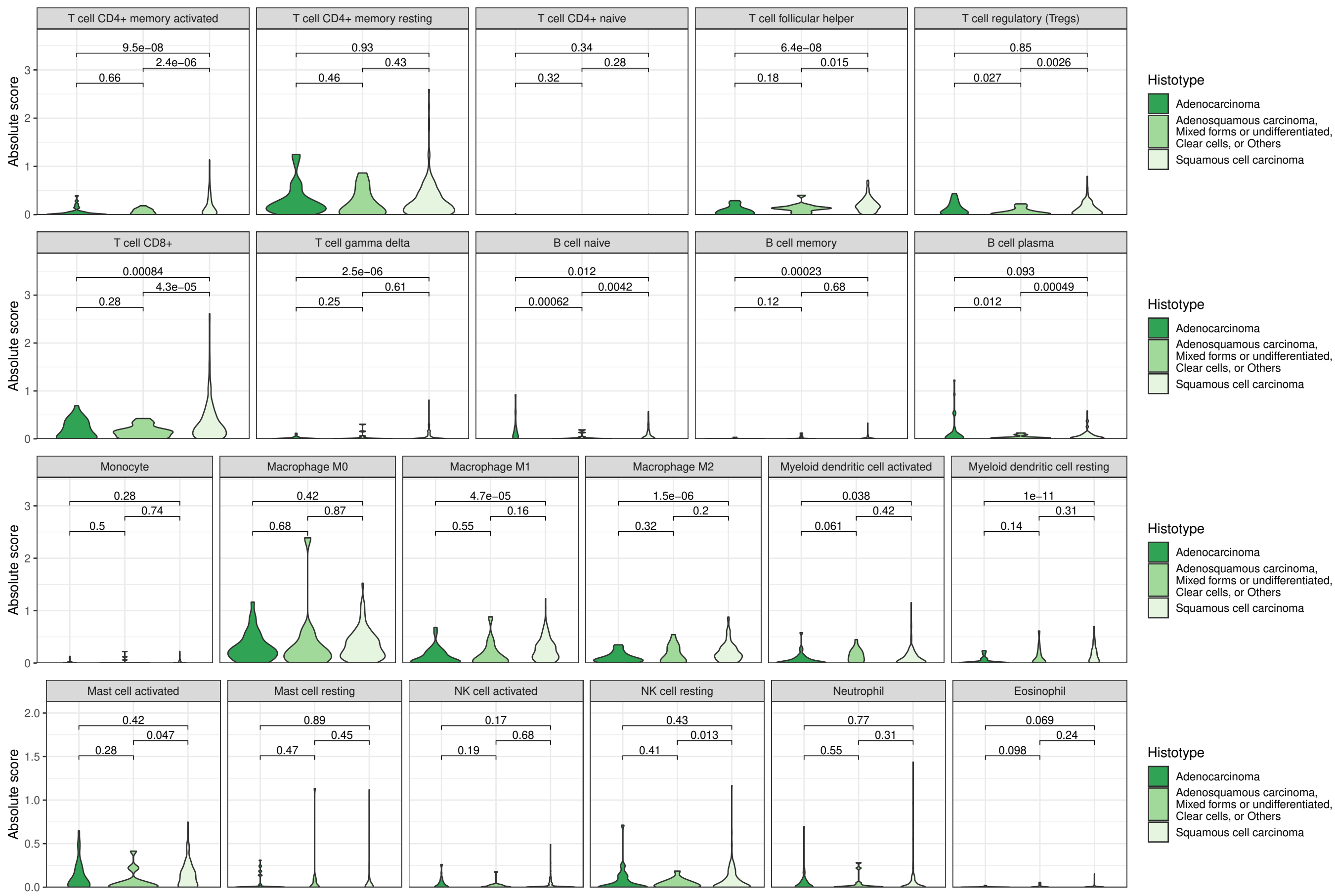

Supplementary Figure 8A. Violin plots show the distribution of immune cell fractions estimated by CIBERSORT (absolute mode) across histotype groups (adenocarcinoma vs. adenosquamous carcinoma vs. squamous cell carcinoma). Differences between groups were evaluated using t-tests.

B

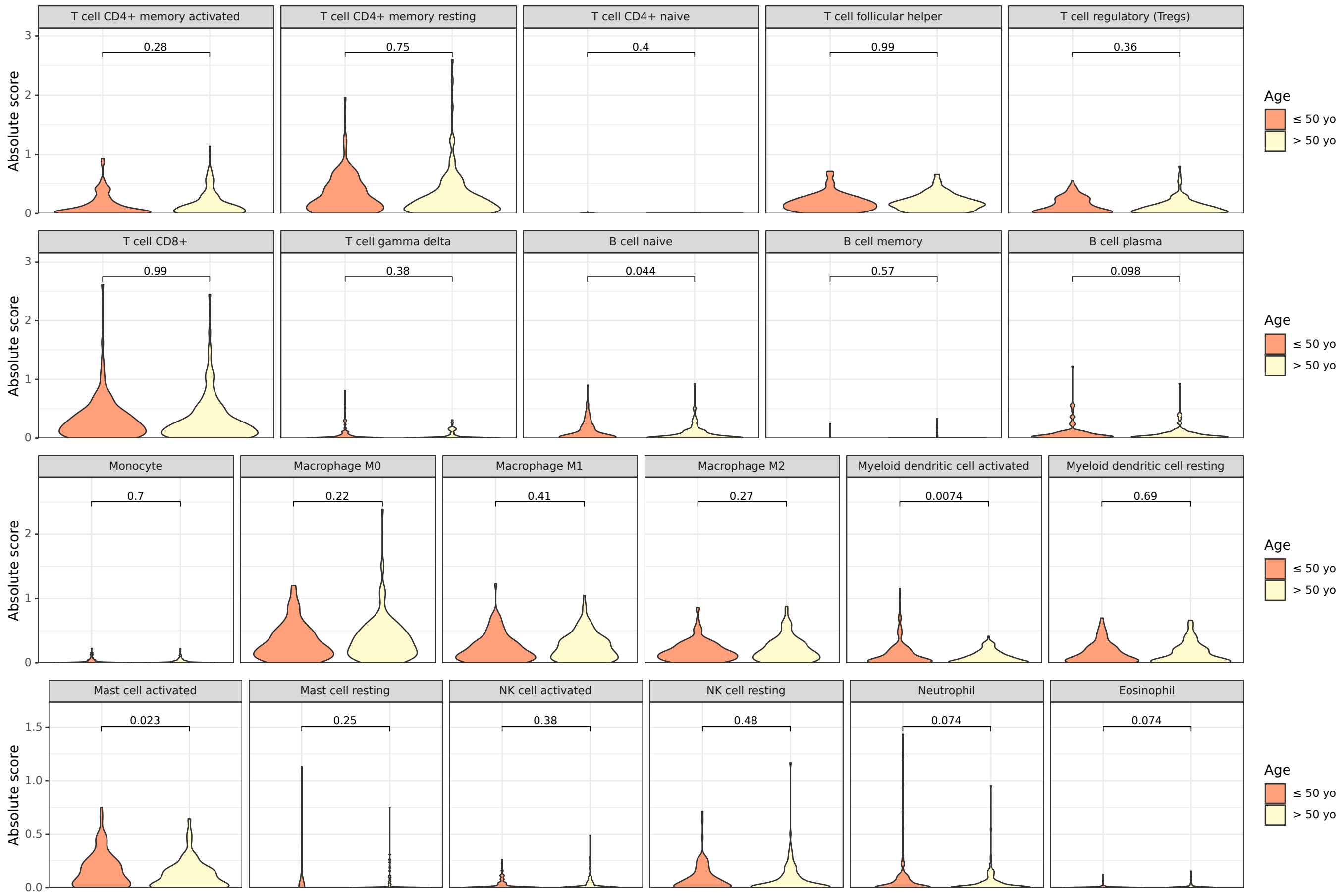

Supplementary Figure 8B. Violin plots show the distribution of immune cell fractions estimated by CIBERSORT (absolute mode) across patient age groups (≤50 vs. > 50 years old). Differences between groups were evaluated using t-tests.

C

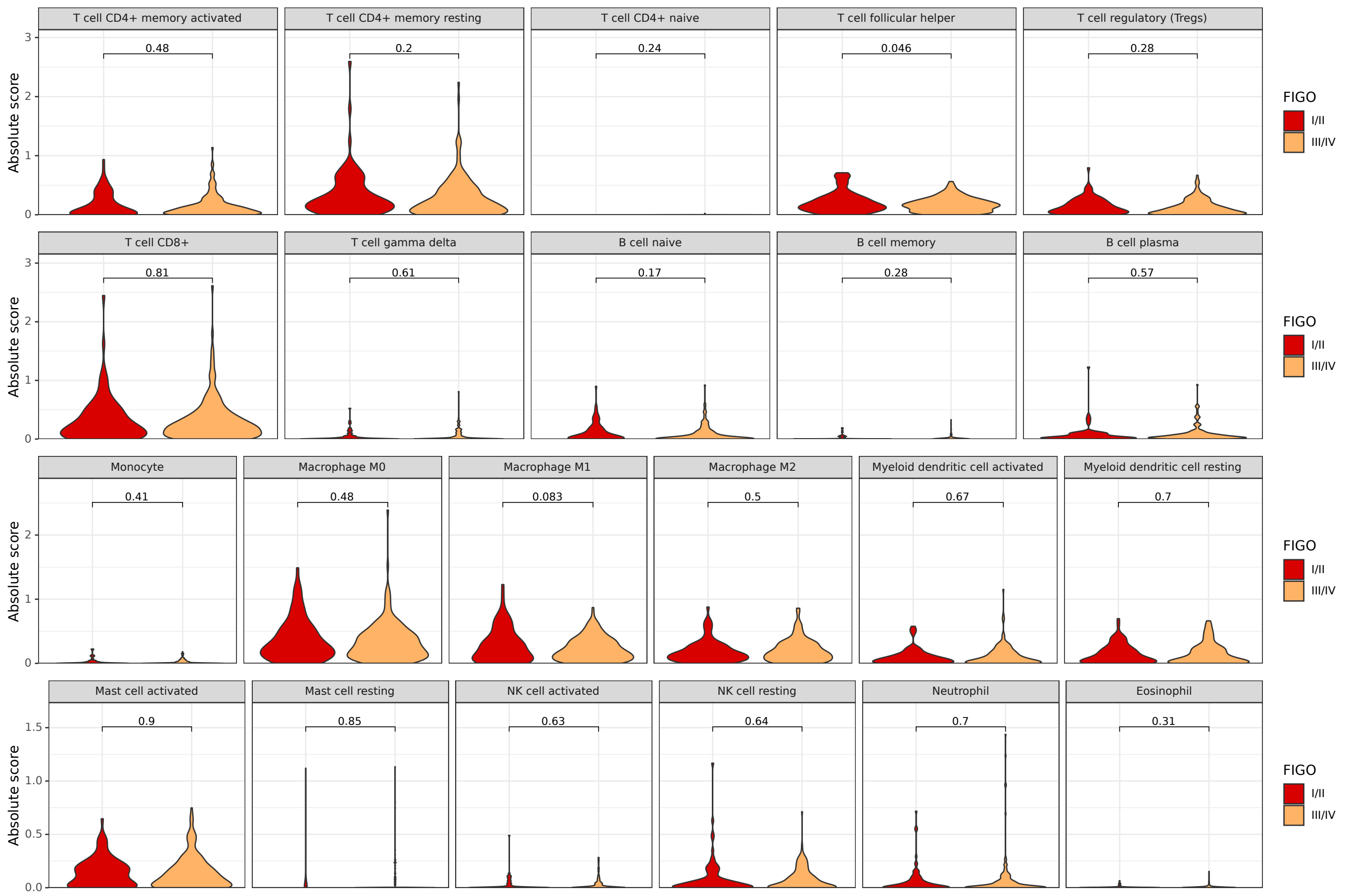

Supplementary Figure 8C. Violin plots show the distribution of immune cell fractions estimated by CIBERSORT (absolute mode) across FIGO stage groups (I, II vs. III, IV). Differences between groups were evaluated using t-tests.

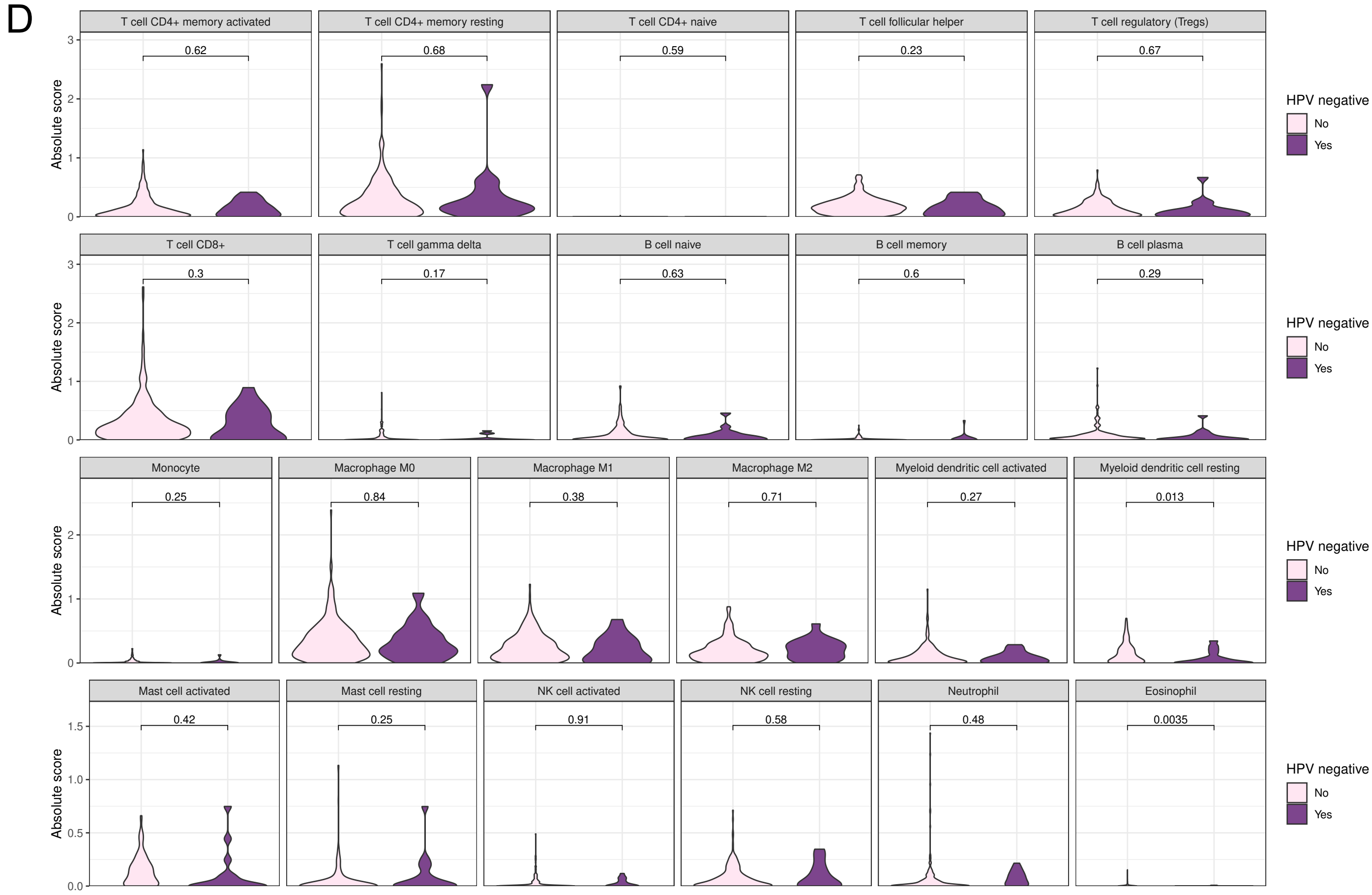

Supplementary Figure 8D. Violin plots show the distribution of immune cell fractions estimated by CIBERSORT (absolute mode) across HPV negative status groups (No vs. Yes). Differences between groups were evaluated using t-tests.

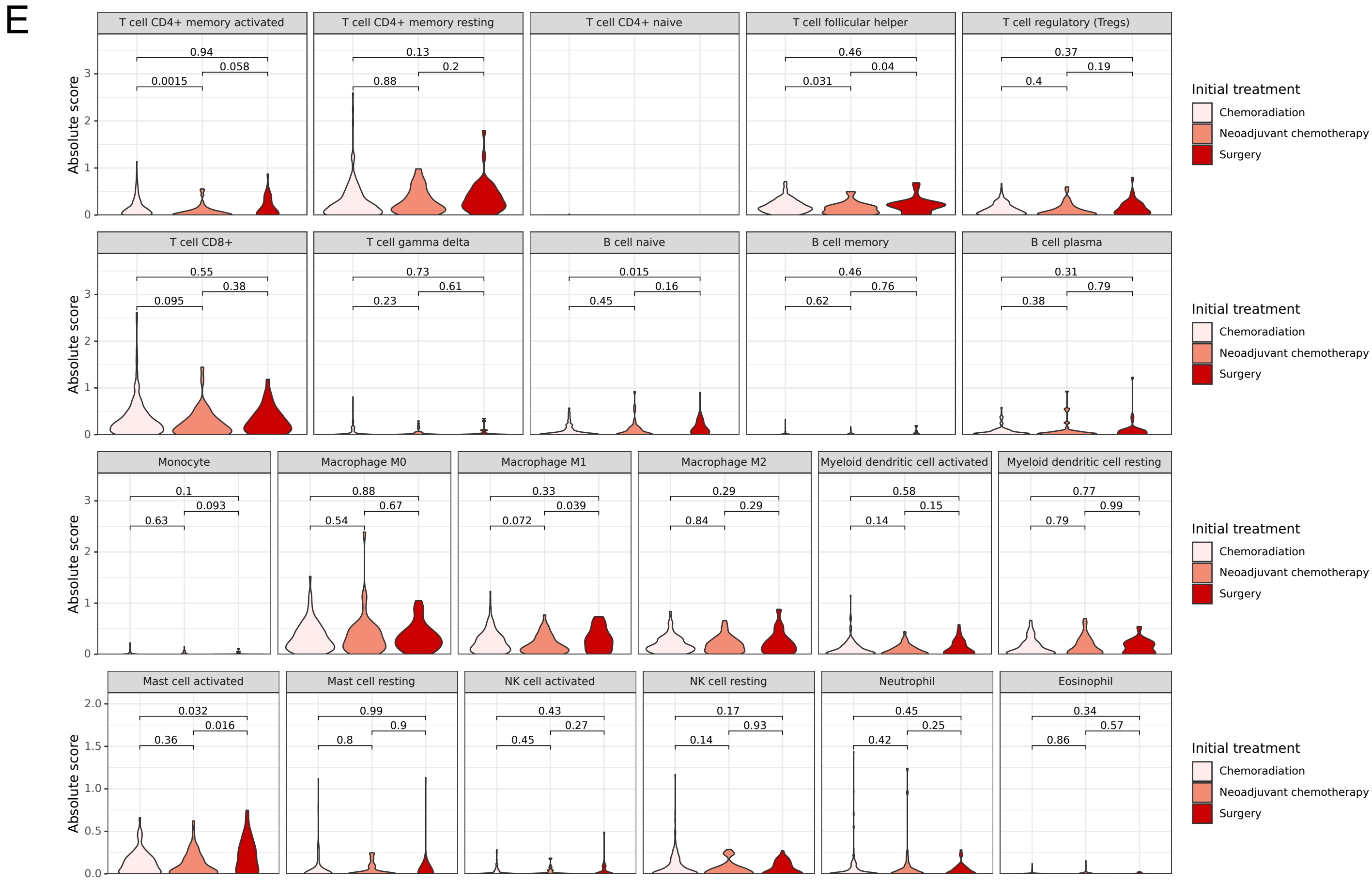

Supplementary Figure 8E. Violin plots show the distribution of immune cell fractions estimated by CIBERSORT (absolute mode) across initial treatment groups (chemoradiation vs. neoadjuvant chemotherapy vs. surgery). Differences between groups were evaluated using t-tests.

F

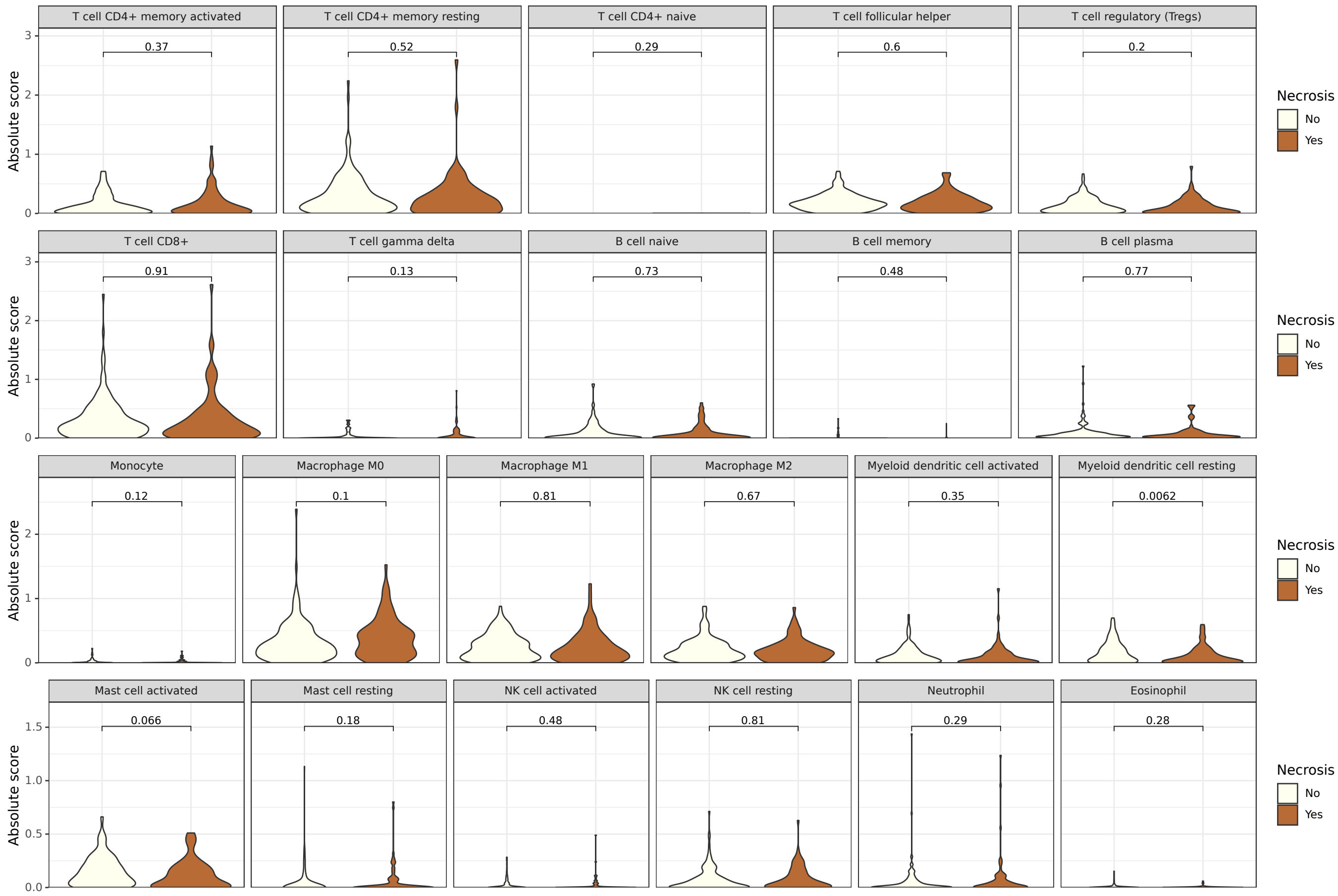

Supplementary Figure 8F. Violin plots show the distribution of immune cell fractions estimated by CIBERSORT (absolute mode) across necrosis groups (absence of necrosis vs. presence of necrosis). Differences between groups were evaluated using t-tests.

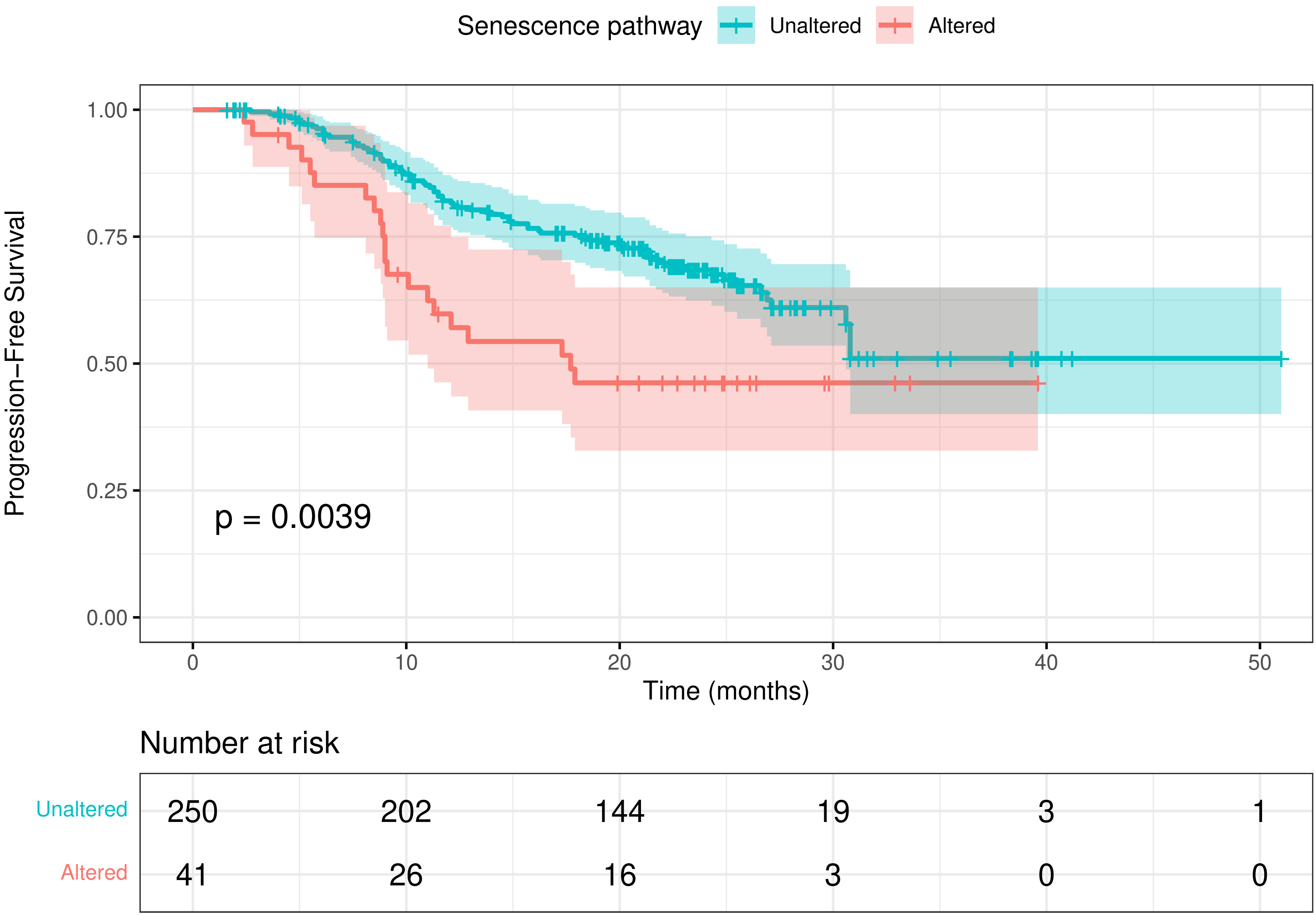

Supplementary Figure 9. Survival curve based on Kaplan-Meier and log-rank test for Senescence pathway

Supplementary Data 1. Matrix of somatic alterations on gene with altered frequency >= 3

Supplementary Data 2. Validated fusions

Supplementary Data 3. MSI and TMB status

Supplementary Data 4. Most contributing SBS signature per tumor

Supplementary Data 5. ShallowHRD results from sWGS datasets

Supplementary Data 6. Correlation between HRD status assessed by sWGS and somatic alterations of HR genes

Supplementary Data 7. Correlation between HRD status assessed by sWGS and germinal alterations of HR genes

Supplementary Data 8. List of signaling pathway and corresponding genes

Supplementary Data 9. Chi2 test results on gene alterations between patients with PFS time < 12 or >= 12 months

Supplementary Data 10. Chi2 test results on pathway alterations between patients with PFS time < 12 or >= 12 months

Supplementary Data 11. Univariable and multivariable Cox models with all clinical features and selected omics variables in the CoxBoost analysis.
